# Differential resilience of chickpea’s reproductive organs to cold stress across developmental stages: insights into antioxidant strategies for enhanced fertility

**DOI:** 10.3389/fpls.2025.1545187

**Published:** 2025-04-07

**Authors:** Deeksha Padhiar, Sarbjeet Kaur, Uday Chand Jha, P. V. Vara Prasad, Kamal Dev Sharma, Sanjeev Kumar, Swarup Kumar Parida, Kadambot H. M. Siddique, Harsh Nayyar

**Affiliations:** ^1^ Department of Botany, Panjab University, Chandigarh, India; ^2^ Crop Improvement Division, Indian Institute of Pulses Research, Kanpur, India; ^3^ Sustainable Intensification Innovation Lab, Kansas State University, Manhattan, KS, United States; ^4^ Department of Agriculture Biotechnology, Chaudhary Sarwan Kumar (CSK) Himachal Pradesh Agricultural University, Palampur, India; ^5^ Department of Plant Sciences, Central University of Punjab, Bhatinda, India; ^6^ Department of Biotechnology, National Institute of Plant Genome Research (NIPGR), New Delhi, India; ^7^ The University of Western Australia (UWA) Institute of Agriculture, The University of Western Australia, Perth, WA, Australia

**Keywords:** *Cicer arietinum*, genotypes, low temperature, anthers, ovules, pollen, pods, seeds

## Abstract

Chickpea is highly sensitive to cold stress during its reproductive stages, leading to significant reductions in potential pod formation due to decreased reproductive success. This study aimed to investigate the specific responses of anthers and ovules to cold stress, explore the role of oxidative stress and antioxidant mechanisms, and understand the relationship between oxidative stress and reproductive function to enhance our understanding of chickpea responses to cold stress. Chickpea seeds of contrasting genotypes—cold-tolerant (ICC 17258, ICC 16349) and cold-sensitive (ICC 15567, GPF 2)—were sown outdoors in early November under optimal conditions (25.5/15.4°C mean day/night temperatures). At 50 days after sowing, plants were subjected to 13/7°C cold stress (12 h light/dark in walk-in growth chambers. Cold stress significantly increased membrane damage and reduced cellular viability in anthers and ovules, particularly in cold-sensitive (CS) genotypes. Oxidative damage was more pronounced in anthers, particularly at anthesis (stage 2), as indicated by elevated malondialdehyde and hydrogen peroxide levels. Cold-tolerant (CT) genotypes exhibited increased antioxidant activity under stress, especially at pre-anthesis (stage 1), followed by declines at later stage, although responses varied by genotype. Anthers exhibited higher overall antioxidants activity than ovules, while ovules demonstrated notably high catalase activity. Among the antioxidants studied, ascorbate peroxidase and glutathione reductase were most prominent in the CT genotype, along with higher levels of ascorbate (AsA) and glutathione (GSH), highlighting the critical role of the AsA–GSH cycle in conferring cold tolerance to chickpea. Exogenous supplementation with 1 mM ascorbate (AsA) and glutathione (GSH) significantly stimulated pollen germination in cold-stressed plants under *in vitro* conditions, with a greater effect observed in CS genotypes. Furthermore, antioxidant activity strongly correlated with key reproductive traits such as pollen germination and ovule viability. This study revealed that the anthers and ovules exhibited distinct responses to cold stress, with significant genotypic differences across key reproductive stages. These insights provide a deeper understanding of cold tolerance mechanisms in chickpea and provide vital clues for breeding strategies to enhance resilience and reproductive success under cold stress.

## 1 Introduction

Cold stress significantly affects plant growth and development, particularly in tropical-origin crops, by disrupting various vegetative and reproductive stages (Croser et al., 2003; Clarke et al., 2004). Chickpea (*Cicer arietinum* L.), an important legume crop providing essential proteins and nutrients to millions worldwide (Bar-El-Dadon et al., 2017), is especially vulnerable to cold stress during the reproductive phase. This vulnerability often results in flower and pod abortion, leading to 30–40% yield losses in cold-sensitive genotypes (Srinivasan et al., 1999; Kiran et al., 2021; Berger et al., 2023). Exposure to temperatures below 15°C has been linked to flower abortion and reduced pod production (Nayyar et al., 2005; Bhandari et al., 2020; Rani et al., 2020; Kaur et al., 2022).

Reproductive development is highly sensitive to cold stress, affecting both male and female reproductive tissues (Thakur et al., 2010; Soualiou et al., 2022). In male organs, low temperatures disrupt tapetum development, delay programmed cell death, and reduce pollen viability, leading to sterility (Liu et al., 2019; Huang et al., 2022). Cold stress also interferes with meiosis and gametogenesis, processes essential for viable pollen production (Liu et al., 2019). Cold stress affects gynoecium development and function in female reproductive structures, although this has been less studied (Albertos et al., 2019). In chickpea, chilling temperatures during the reproductive phase can cause floral abortion, reduce pollen viability, inhibit stigma receptivity, and impair pollen tube growth, resulting in fertilization failure (Clarke et al., 2004; Kumar et al., 2010; Berger et al., 2012).

The detrimental impacts of cold stress on reproductive development are not unique to chickpea. Similar impacts have been documented in other crops, including wheat (Chakrabarti et al., 2011), rice (Arshad et al., 2017; González-Schain et al., 2019), and soybean (Gass et al., 1996). For example, low temperatures can inhibit pollen tube growth and reduce pollen viability, decreasing pod set (Pacini and Dolferus, 2019) in rice (*Oryza sativa*; Yamamori et al., 2021) and chickpea (Rani et al., 2020). Such impacts on reproductive success have been attributed to disruptions in carbohydrate metabolism (Kiran et al., 2021) and hormonal imbalances (Nikkhoye-Tanha et al., 2024), further illustrating the widespread effects of cold stress on plant fertility.

Understanding the physiological mechanisms that govern these responses in chickpea is critical for developing effective strategies to mitigate cold stress effects. A major physiological consequence of cold stress is the excessive accumulation of reactive oxygen species (ROS), leading to oxidative damage (Garcia-Caparros et al., 2021), such as lipid peroxidation, protein denaturation, and cellular dysfunction (Mittler et al., 2022). These effects impair vital processes such as photosynthesis and respiration, ultimately reducing plant productivity (Baek and Skinner, 2012; Dreyer and Dietz, 2018; Soualiou et al., 2022). Studies indicate that cold-sensitive chickpea genotypes exhibit elevated oxidative stress markers and reduced fertility when exposed to chilling conditions (Thakur et al., 2020; Adhikari et al., 2022).

Comparing the oxidative stress responses of anthers and ovules is particularly important, as these structures serve distinct roles in reproduction. Anthers are critical for male gamete formation, whereas ovules are essential for female gamete development. Previous studies have shown that temperature fluctuations differentially impact the functionality of male and female reproductive structures (Albertos et al., 2019; Liu et al., 2019). Understanding how these structures cope with oxidative damage under cold stress can provide valuable insights into chickpea resilience.

Plants have evolved complex antioxidant defense mechanisms to combat ROS-induced damage during cold stress (Gusain et al., 2023; Jahed et al., 2023). These mechanisms include enzymatic antioxidants such as ascorbate peroxidase (APX) and glutathione reductase (GR) and non-enzymatic antioxidants like ascorbate (AsA) and reduced glutathione (GSH) that neutralize ROS and stabilize cellular membranes, preserving pollen and ovule functionality during critical developmental stages. Despite their importance, the roles of these antioxidants in protecting chickpea’s reproductive tissues at different developmental stages, such as pre-anthesis and anthesis, which remain underexplored. Evaluating these stages can reveal when reproductive tissues are most vulnerable to cold-induced damage, which is crucial for developing targeted strategies to enhance chickpea cold tolerance through breeding programs.

Information on the relative sensitivity of anthers and ovules to cold stress during development, especially in the management of oxidative stress, is lacking. Therefore, this study was planned to investigate the effects of cold stress on oxidative damage in chickpea’s reproductive structures, focusing on anthers and ovules at pre-anthesis and anthesis stages. By exploring genotypic differences in oxidative stress management and linking oxidative stress responses to key reproductive traits, the study aimed to provide vital insights into enhancing chickpea’s resilience and fertility under cold conditions.

## 2 Materials and methods

### 2.1 Raising of plants and cold stress treatment

Seeds of contrasting chickpea genotypes—cold-tolerant (ICC 17258 and ICC 16349) and cold-sensitive (ICC 15567 and GPF 2) were soaked for 12 hours and inoculated with a suitable *Mesorhizobium ciceri* sp. culture (2.0 g kg^−1^ seed) before sowing. The contrasting chickpea genotypes were selected based on our preliminary screening studies, which involved evaluating 40 genotypes for reproductive cold tolerance (Pod set) under controlled environments (13/7°C). The genotypes exhibiting the most contrasting levels of pod set (high and low) were then selected for this more detailed study. Five inoculated seeds were planted in each pot containing 8 kg of a sandy loam and farmyard manure mixture (3: 1 ratio), enriched with tricalcium phosphate fertilizer (10 mg kg^-^¹ soil). Fifteen days after sowing (DAS), plants were thinned to two per pot. The plants were initially grown outdoors in November under natural environmental conditions, protected from bird and animal interference by wire enclosures. Weather conditions included mean day/night temperatures of 25.5/15.4°C, 1,300–1,500 μmol m^-^² s^-^¹ light intensity, and 60–70% relative humidity (**Supplementary Figure S1** **).**

At 50 days after sowing, the plants were transferred to walk-in growth chambers for controlled treatments. Cold stress was imposed with a temperature of 13/7°C (12 h light/12 h dark), 600 μmol m^-^² s^-^¹ light intensity, and 65–70% relative humidity. The diurnal temperature gradient was reduced by 1°C per day until reaching the target temperature, maintained through podding (15 days), and subsequently increased by 2°C per day to attain 30/23°C (12 h day/night) until maturity. Control plants were kept under standard environmental conditions (25/15°C) until podding and then subjected to similar temperature increases to reach 30/23°C (12 h day/night) until maturity. Anther and ovule samples were collected at two-time points corresponding to pre-anthesis (5 mm bud size, flower stage 12; S1) and anthesis (10 mm flower size, flower stage 18; S2) to assess the effects of continuous cold stress on reproductive organs, following the developmental staging described by Kiran et al. (2019). These samples were analyzed for the traits described below.

### 2.2 Electrolyte leakage

Electrolyte leakage (EL), indicative of membrane damage, was assessed in anthers and ovules. Samples were washed with deionized water, cut into small pieces, and incubated in 10 mL of deionized water at 25°C for 12 hours. Initial electrical conductivity (C1) was measured, followed by heating the tissue at 80°C in a water bath for 10–15 minutes to record the final electrical conductivity (C2). Membrane damage was calculated as EL%: (C1/C2) × 100 (Kaushal et al., 2013).

### 2.3 Cellular viability

Cellular viability was assessed using 2,3,5-triphenyl tetrazolium chloride (TTC), transforming the colorless solution into a dark red formazan through cellular reduction. Anthers and ovules were collected and incubated in a solution containing TTC (500 mg per 100 mL) and 50 mM sodium phosphate (pH 7.4) at 25°C in the dark for 1 hour without agitation. After incubation, the tissues were extracted twice using 95% ethanol, and the extracts were combined to make a final volume of 10 mL. Absorbance of the resultant crimson solution was measured at 530 nm using a spectrophotometer. Cellular viability was expressed as absorbance per gram of fresh weight (Steponkus and Lanphear, 1967).

### 2.4 Malondialdehyde

Malondialdehyde (MDA) content, an indicator of lipid peroxidation, was quantified by homogenizing fresh tissues in 0.1% trichloroacetic acid (TCA), centrifuging at 3,360 *g* for 5 minutes, and reacting the supernatant with 4 mL thiobarbituric acid (0.5%) in 20% TCA. The reaction mixture was heated at 95°C for 30 minutes, cooled in an ice bath, and centrifuged at 3,360 *g* for 10 minutes (4°C). Absorbance was measured at 532 nm, and MDA content was calculated using an extinction coefficient of 155 mM cm^−1^, reported as nmol g^−1^ dry weight (DW) (Heath and Packer, 1968).

### 2.5 Hydrogen peroxide concentration

The hydrogen peroxide (H_2_O_2_) concentration in anthers and ovules was determined by extracting fresh samples in 5 mL of chilled 80% acetone and filtered using Whatman filter paper. The filtrate was mixed with 4 mL of titanium reagent and 5 mL of 25% ammonia solution, followed by centrifugation at 3,360 *g* for 10 minutes. The resulting pellet was dissolved in 1 M H_2_SO_4_ before measuring the optical density at 410 nm. The H_2_O_2_ concentration was calculated using an extinction coefficient of 0.28 mmol cm^−1^, expressed as nanomoles per gram of DW (Mukherjee and Choudhuri, 1983).

### 2.6 Enzymatic antioxidants

#### 2.6.1 Superoxide dismutase

Superoxide dismutase (SOD) activity was quantified by extracting fresh tissue in a cold 50 mM phosphate buffer (pH 7.0) and centrifuging at 3,360 *g* for 5 minutes (4°C). The reaction mixture contained 0.1 mL enzyme extract, 50 mM phosphate buffer (pH 7.8), 13 mM methionine, 25 mM nitroblue tetrazolium chloride (NBT), and 0.1 mM ethylene diamine tetraacetic acid (EDTA), making a total volume of 3 mL. To initiate the reaction, 2 mM riboflavin was added before exposing the mixture to a 15 W fluorescent light for 10 minutes. Absorbance was measured at 560 nm, and SOD activity was recorded and expressed as units per mg of protein (Dhindsa and Matowe, 1981).

#### 2.6.2 Catalase

The tissue was extracted as per SOD activity using a reaction mixture comprising 0.1 mL enzyme extract, 50 mM phosphate buffer (pH 7), and 200 mM H_2_O_2_. Optical density was measured at 410 nm over 3 minutes. Catalase activity was calculated using an extinction coefficient of 40 mM cm^−1^, expressed as mmol H_2_O_2_ decomposed per mg of protein (Teranishi et al., 1974).

#### 2.6.3 Ascorbate peroxidase

Ascorbate peroxidase (APX) activity was measured by adding 0.1 mL enzyme extract to a reaction mixture containing 50 mM phosphate buffer, 0.5 mM ascorbic acid, and 0.1 mM EDTA, with H_2_O_2_ as the substrate. Catalytic activity was calculated using an extinction coefficient of 2.8 mM cm^−1^, expressed as mmol of oxidized donor decomposed per minute per mg of protein (Nakano and Asada, 1981).

#### 2.6.4 Glutathione reductase

Glutathione reductase (GR) activity was determined by mixing 0.1 mL of enzyme extract with a reaction mixture containing 1.5 mL phosphate buffer (0.1 M, pH 7.6), 0.2 mL bovine serum albumin (BSA), 0.35 mL nicotinamide adenine dinucleotide phosphate (NADP), and 0.1 mL oxidized glutathione. The reduction in absorbance was measured at 340 nm over 3 minutes, with GR activity expressed as mmol of oxidized donor decomposed per minute per mg of protein (Mavis and Stellwagen, 1968).

### 2.7 Non-enzymatic antioxidants

#### 2.7.1 Ascorbic acid

Ascorbic acid (AsA) content was determined by extracting fresh tissue in 6% TCA, followed by centrifugation at 3,649.15 *g* for 15 minutes. To 4 mL of supernatant, 2 mL of 2% dinitrophenylhydrazine (DNPH) and a drop of 10% thiourea were added. The assay mixture was heated in a water bath for 15 minutes and cooled to room temperature. After cooling, 5 mL of cold sulfuric acid was added, and the optical density was measured at 530 nm. The AsA content was calculated using a standard curve and reported as mg per gram of DW (Mukherjee and Choudhuri, 1983).

#### 2.7.2 Reduced glutathione

Reduced glutathione (GSH) content was measured by homogenizing fresh tissue in 2 mL metaphosphoric acid and centrifuging at 3,650 *g* for 15 minutes before adding 0.6 mL of 10% sodium citrate to the resulting supernatant (0.9 mL). The reaction mixture contained 100 μL extract, 100 μL distilled water, 100 μL of 6 mM 5,5-dithio-bis-(2)-nitrobenzoic acid (DTNB), and 700 μL of 0.3 mM NADPH. Finally, 10 μL of GR was added to the mixture. The optical density was measured at 412 nm, and GSH content was calculated using a standard curve and expressed as nmol per gram of DW (Griffith, 1980).

### 2.8 Floral biology

#### 2.8.1 Pollen germination

Pollen germination was assessed by collecting anthers from flowers of cold-treated plants and tapping them to release pollen grains onto slides. The pollen germination was then evaluated in a growth medium containing 10% sucrose, 990 mM nitrate (pH 6.5), 1,640 mM boric acid, 812 mM magnesium sulfate, and 1,269 mM calcium nitrate. The percentage of germinated pollen was calculated (Brewbaker and Kwack, 1963; Shivanna and Rangaswamy, 1992; Kaushal et al., 2013).

#### 2.8.2 Pollen viability

Pollen viability was assessed by dissecting fully opened and functional flowers from cold-treated plants to collect anthers. The anthers were tapped onto a slide, releasing 100–200 pollen grains, stained with 0.5% acetocarmine, and examined for size, shape, and color intensity under a microscope. Viability was presented as a percentage (Kaushal et al., 2013).

#### 2.8.3 Stigma receptivity

Stigma receptivity was evaluated using the esterase test (Mattsson et al., 1974). Stigmas were collected one day before anthesis and soaked in a solution containing naphthaleneacetic acid (α-NAA) and Fast Blue B prepared in phosphate buffer at 37°C for 15 minutes. Stigma receptivity was assessed by the intensity of the brown color that developed, rated on a scale from 1 to 5, with 1 indicating minimal receptivity and 5 indicating maximum receptivity.

#### 2.8.4 Ovule viability

Ovules were collected from flowers and evaluated for viability using a TTC reduction assay. They were placed on a glass slide and treated with 0.5% TTC (prepared from a 1% solution). The slides were then transferred to a Petri dish containing filter paper moistened with distilled water and incubated in a chamber at 25°C for 15 minutes. The resulting red color was rated on a scale from 1 to 5, with 1 indicating minimal receptivity and 5 indicating maximum receptivity (Kaushal et al., 2013).

#### 2.8.5 Effect of ascorbate and glutathione on pollen germination (*in vitro*)

Pollen grains, collected from control and stressed plants of both the genotypes, were germinated (as per the method described in 2.8.1.) in a growth medium supplemented with ascorbate or glutathione (1 mM). The germination percentage was calculated following the method given in section 2.8.1.

### 2.9 Experimental design and statistical analysis

The experiment had a two-factor randomized block design, including cold-tolerant and cold-sensitive chickpea genotypes exposed to two distinct treatment conditions. Each genotype was represented by nine pots (two plants per pot) and three replicates per treatment. For yield trait measurements, nine pots (three pots in triplicate) were maintained separately for each treatment. The pots were rearranged periodically throughout the experiment to reduce positional effects. We used analysis of variance (ANOVA) with the stats package version 4.3.0 in R Studio (R Core Team, 2023), gvlma package to analyze how genotype, treatment, replication, and genotype-by-treatment interaction affected traits at low temperatures (Pena and Slate, 2019). Genotypic differences within each treatment were analyzed using one-way ANOVA followed by Tukey’s HSD test using package multcompView (Graves et al., 2024). Treatment differences (cold *vs*. control) within each genotype were analyzed using a paired t-test. The packages ggplot2, factoextra, and factoMineR in R Studio were used to do principal component analyses (PCAs) (Wickham, 2016; Kassambara and Mundt, 2020; Lê et al., 2008). To make the Correlation plot, the Corrplot package version 0.95 in R Studio was used (Wei and Simko, 2017). 2-way ANOVA results are summarized in **Supplementary Table S1**.

## 3 Results

### 3.1 Membrane damage

Cold stress significantly intensified membrane damage (as electrolyte leakage) in both anthers and ovules of cold-tolerant (CT) and cold-sensitive (CS) genotypes relative to control conditions (**Supplementary Figure S2**) Specifically, in anthers **Supplementary Figures S2a, b**) CT genotypes exhibited a 36% increase at pre-anthesis and a 48–69% increase at anthesis while CS genotypes exhibited a 73–76% increase at stage 1, escalating to 147–157% at stage 2. In ovules (**Supplementary Figures S2c, d**) CT genotypes increased membrane damage by 17–28% at stage 1 and 31–36% at stage 2, while CS genotypes experienced a 46–53% increase at stage 1 and 89–115% at stage 2.

### 3.2 Cellular viability

Cold stress effects on cellular viability varied between developmental stages (**Supplementary Figure S3**). At stage 1 (**Supplementary Figures S3a, c**), CT genotypes exhibited a 10–16% increase in anther viability and a 29–33% increase in ovule viability compared to controls. In contrast, CS genotypes showed smaller increases of 7–11% in anthers and 21–26% in ovules. At stage 2 (**Supplementary Figures S3b, d**) CT genotypes saw declines of 10–25% (anthers) and 22–24% (ovules), while CS genotypes had steeper declines of 25–40% (anthers) and 20–27% (ovules) compared to controls.

### 3.3 Malondialdehyde

**Figure 1:**
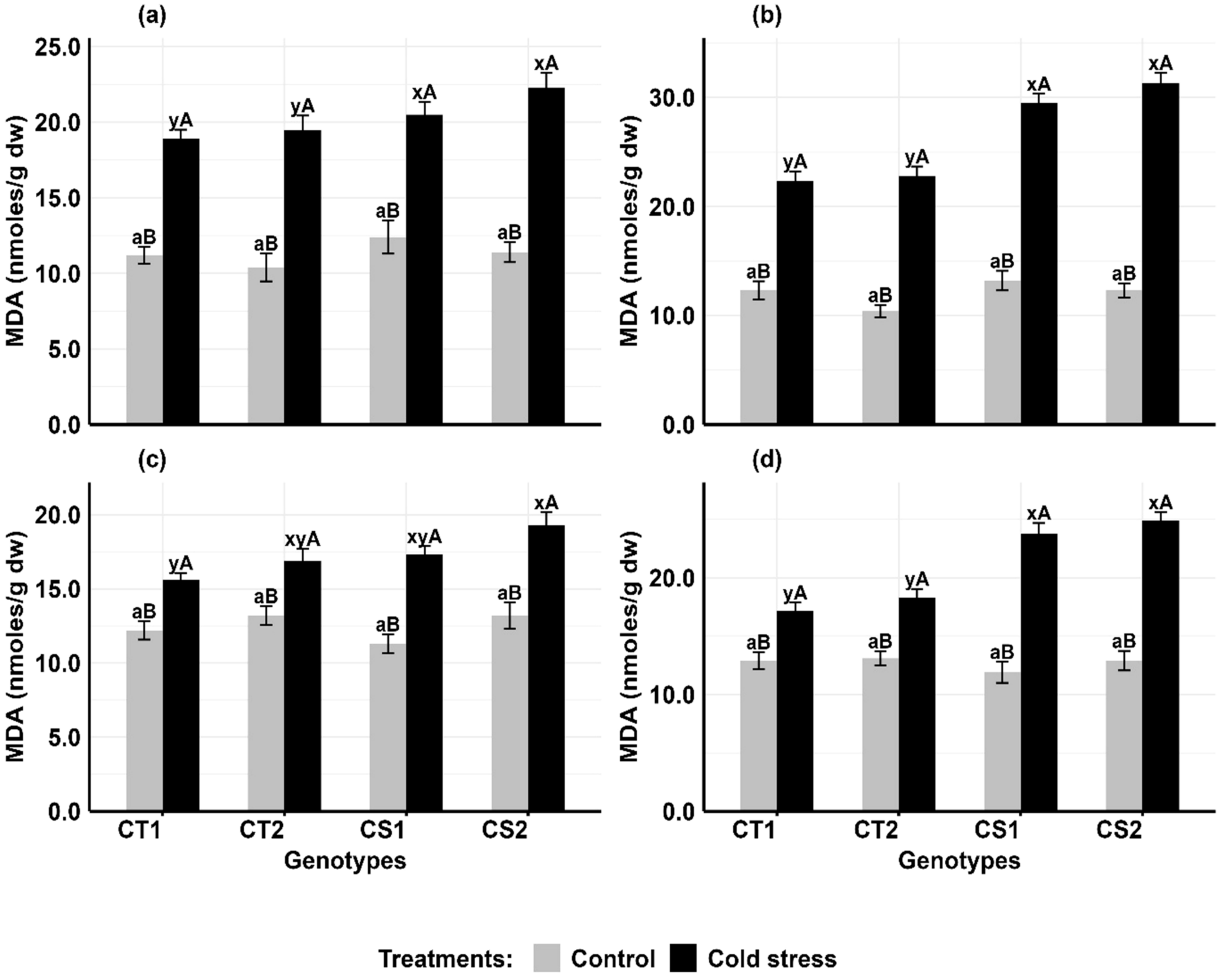
Malondialdehyde (MDA) **(a)** Anthers-stage 1, **(b)** Anthers-stage 2, **(c)** Ovules-stage 1, **(d)** Ovules-stage 2, under control and cold stress conditions. CT1: Cold tolerant genotype 1 (ICC 17258); CT2: Cold tolerant genotype 2 (ICC 16349); CS1: Cold sensitive genotype 1 (ICC 15567); CS2: Cold sensitive genotype 2 (GPF-2). Vertical bars represent standard errors (n=3). Genotypic differences within each treatment were analyzed using one-way ANOVA followed by Tukey's HSD test. Different lowercase letters (a for control and x, y for cold stress) denote significant differences (p < 0.05) among genotypes within the same treatment. Treatment differences (Control vs. Cold Stress) within each genotype were analyzed using a paired t-test. Different uppercase letters **(A, B)** indicate significant differences (p < 0.05) between treatments within the same genotype, where 'A' is indicated for the highest value and 'B' is indicated the smallest value.

Relative to controls, cold stress significantly elevated MDA levels in anthers and ovules of all genotypes, with the most pronounced effects at stage 2 (**Figure 1**). In anthers (**Figures 1a, b**), CT genotypes increased MDA levels by 68–87% at stage 1 and 81–96% at stage 2, while CS genotypes experienced greater increases of 65–95% at stage 1 and 123–154% at stage 2. In ovules (**Figures 1c, d**), CT genotypes increased MDA levels by 28–29% at stage 1 and 33–45% at stage 2, while CS genotypes increased by 46–53% at stage 1 and 93–100% at stage 2.

### 3.4 Hydrogen peroxide

**Figure 2:**
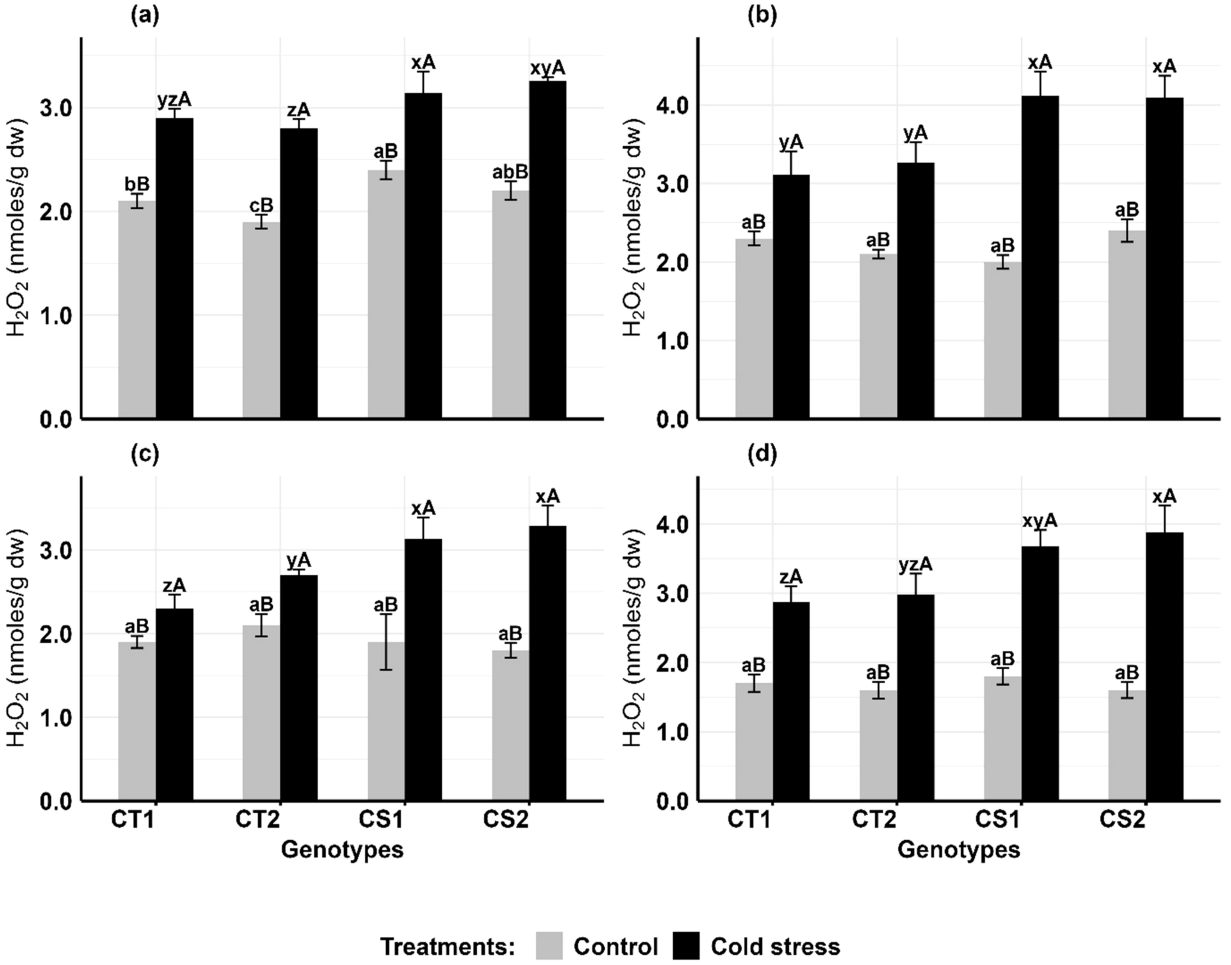
Hydrogen peroxide (H2O2) **(a)** Anthers-stage 1, **(b)** Anthers-stage 2, **(c)** Ovules-stage 1, **(d)** Ovules-stage 2, under control and cold stress conditions. CT1: Cold tolerant genotype 1 (ICC 17258); CT2: Cold tolerant genotype 2 (ICC 16349); CS1: Cold sensitive genotype 1 (ICC 15567); CS2: Cold sensitive genotype 2 (GPF-2). Vertical bars represent standard errors (n=3). Genotypic differences within each treatment were analyzed using one-way ANOVA followed by Tukey's HSD test. Different lowercase letters (a, b, c for control and x, y, z for cold stress) denote significant differences (p < 0.05) among genotypes within the same treatment. Treatment differences (Control vs. Cold Stress) within each genotype were analyzed using a paired t-test. Different uppercase letters (A, B) indicate significant differences (p < 0.05) between treatments within the same genotype, where 'A' is indicated for the highest value and 'B' is indicated the smallest value.

Cold stress significantly elevated hydrogen peroxide concentrations in anthers and ovules of all genotypes, with CS genotypes showing greater susceptibility (**Figure 2**). In anthers (**Figures 2a, b**), CT genotypes increased H_2_O_2_ levels by 38–47% at stage 1 and 52–55% at stage 2, while CS genotypes increased by 50–59% at stage 1 and 91–106% at stage 2 compared to controls. In ovules (**Figures 2c, d**), CT genotypes increased H_2_O_2_ levels by 21–28% at stage 1 and 68–72% at stage 2, while CS genotypes increased by 64–82% at stage 1 and 104–142% at stage 2.

### 3.5 Enzymatic antioxidants

#### 3.5.1 Superoxide dismutase

**Figure 3:**
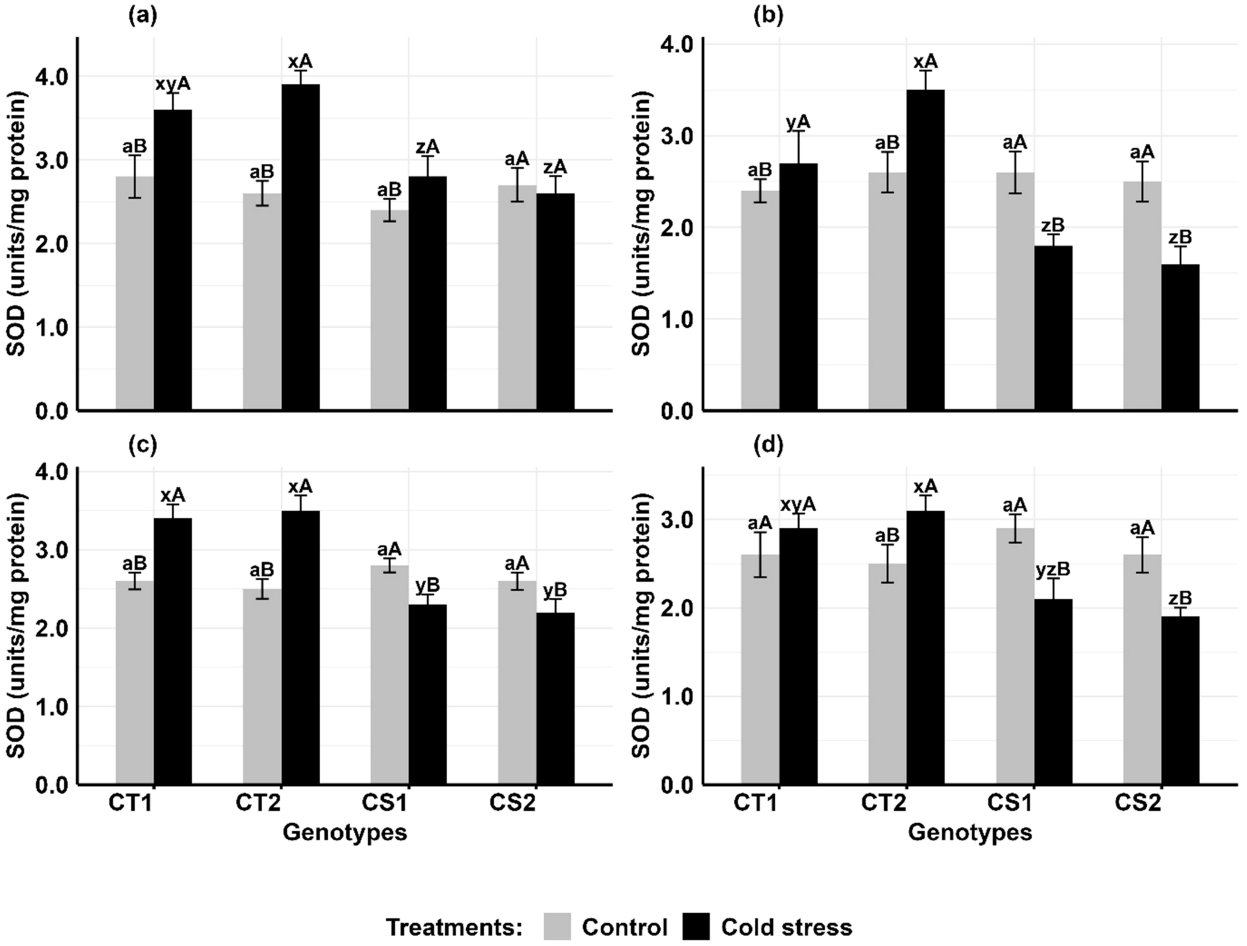
Superoxide dismutase (SOD) **(a)** Anthers-stage 1, **(b)** Anthers-stage 2, **(c)** Ovules-stage 1, **(d)** Ovules-stage 2, under control and cold stress conditions. CT1: Cold tolerant genotype 1 (ICC 17258); CT2: Cold tolerant genotype 2 (ICC 16349); CS1: Cold sensitive genotype 1 (ICC 15567); CS2: Cold sensitive genotype 2 (GPF-2). Vertical bars represent standard errors (n=3). Genotypic differences within each treatment were analyzed using one-way ANOVA followed by Tukey's HSD test. Different lowercase letters (a for control and x, y, z for cold stress) denote significant differences (p < 0.05) among genotypes within the same treatment. Treatment differences (Control vs. Cold Stress) within each genotype were analyzed using a paired t-test. Different uppercase letters (A, B) indicate significant differences (p < 0.05) between treatments within the same genotype, where 'A' is indicated for the highest value and 'B' is indicated for the smallest value.

In comparison to controls, cold stress increased SOD activity in the anthers and ovules of CT genotypes at both developmental stages, while CS genotypes had variable results (**Figure 3**). In anthers (**Figures 3a, b**), SOD activity increased in CT genotypes by 28–46% at stage 1 and 15–37% at stage 2, while CS genotypes exhibited contrasting trends at Stage 1, with one showing a 15% increase and another a 5% decrease followed by a 33–35% decline at stage 2. Similarly, in ovules (**Figures 3c, d**), SOD activity increased in CT genotypes by 30–48% at stage 1 and 10–19% at stage 2, while in CS genotypes, there was a decline of 10–15% at stage 1 and a decline of 26–27% at stage 2.

#### 3.5.2 Catalase

**Figure 4:**
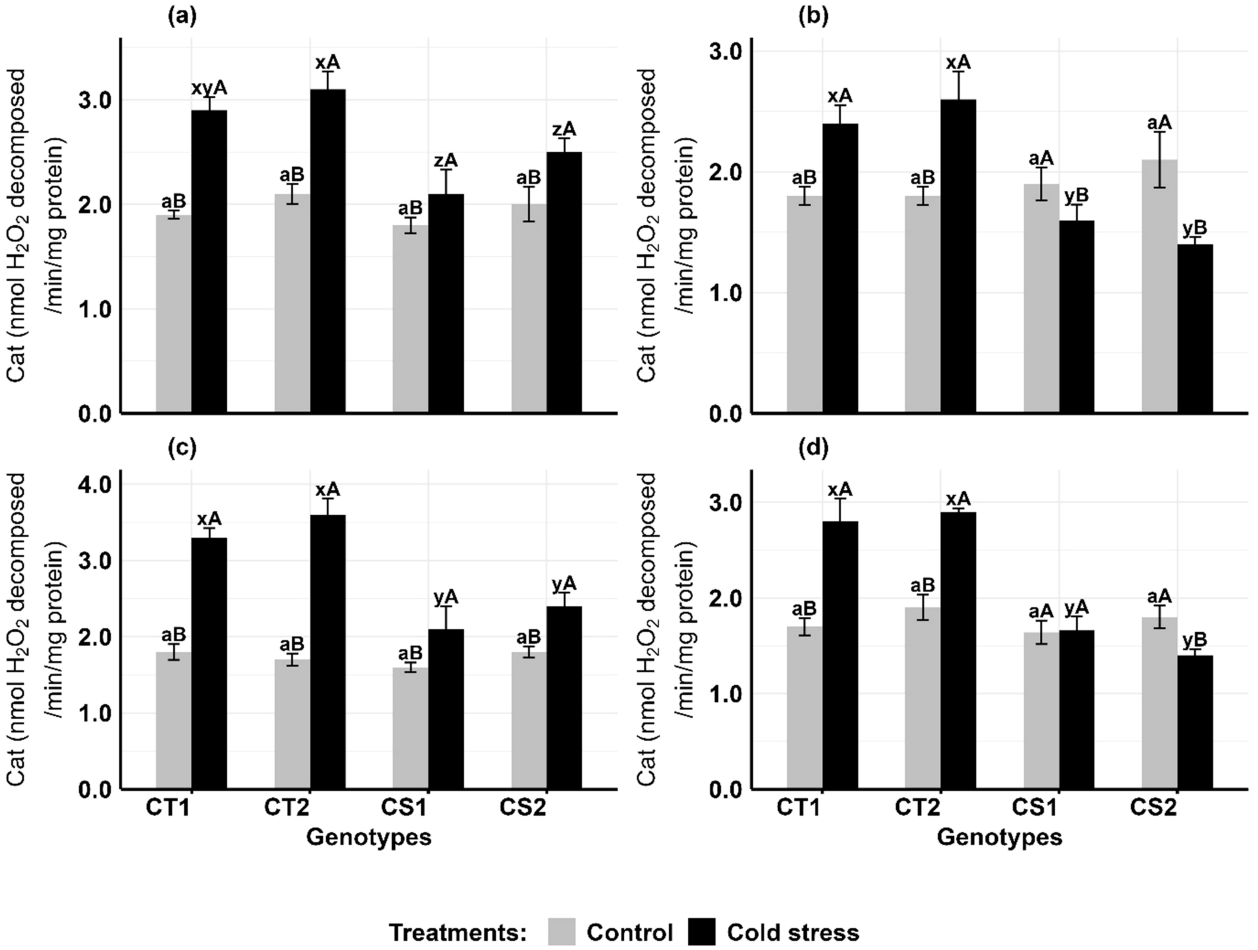
Catalase (Cat) **(a)** Anthers-stage 1, **(b)** Anthers-stage 2, **(c)** Ovules-stage 1, **(d)** Ovules-stage 2, under control and cold stress conditions. CT1: Cold tolerant genotype 1 (ICC 17258); CT2: Cold tolerant genotype 2 (ICC 16349); CS1: Cold sensitive genotype 1 (ICC 15567); CS2: Cold sensitive genotype 2 (GPF-2). Vertical bars represent standard errors (n=3). Genotypic differences within each treatment were analyzed using one-way ANOVA followed by Tukey's HSD test. Different lowercase letters (a for control and x, y, z for cold stress) denote significant differences (p < 0.05) among genotypes within the same treatment. Treatment differences (Control vs. Cold Stress) within each genotype were analyzed using a paired t-test. Different uppercase letters (A, B) indicate significant differences (p < 0.05) between treatments within the same genotype, where 'A' is indicated for the highest value and 'B' is indicated the smallest value.

Cold stress increased catalase activity in the anthers and ovules of CT genotypes at both developmental stages, while CS genotypes had variable results (**Figure 4**). In anthers (**Figures 4a, b**), CAT activity increased in CT genotypes by 47–52% at stage 1 and 33–55% at stage 2, while CS genotypes increased by 16–25% at stage 1 but decreased by 15–35% at stage 2. In ovules (**Figures 4c, d**), CAT activity increased in CT genotypes by 83–111% at stage 1 and 52–64% at stage 2, while CS genotypes increased CAT activity by 31–33% at stage 1 but decreased by 42% at stage 2.

#### 3.5.3 Ascorbate peroxidase

**Figure 5:**
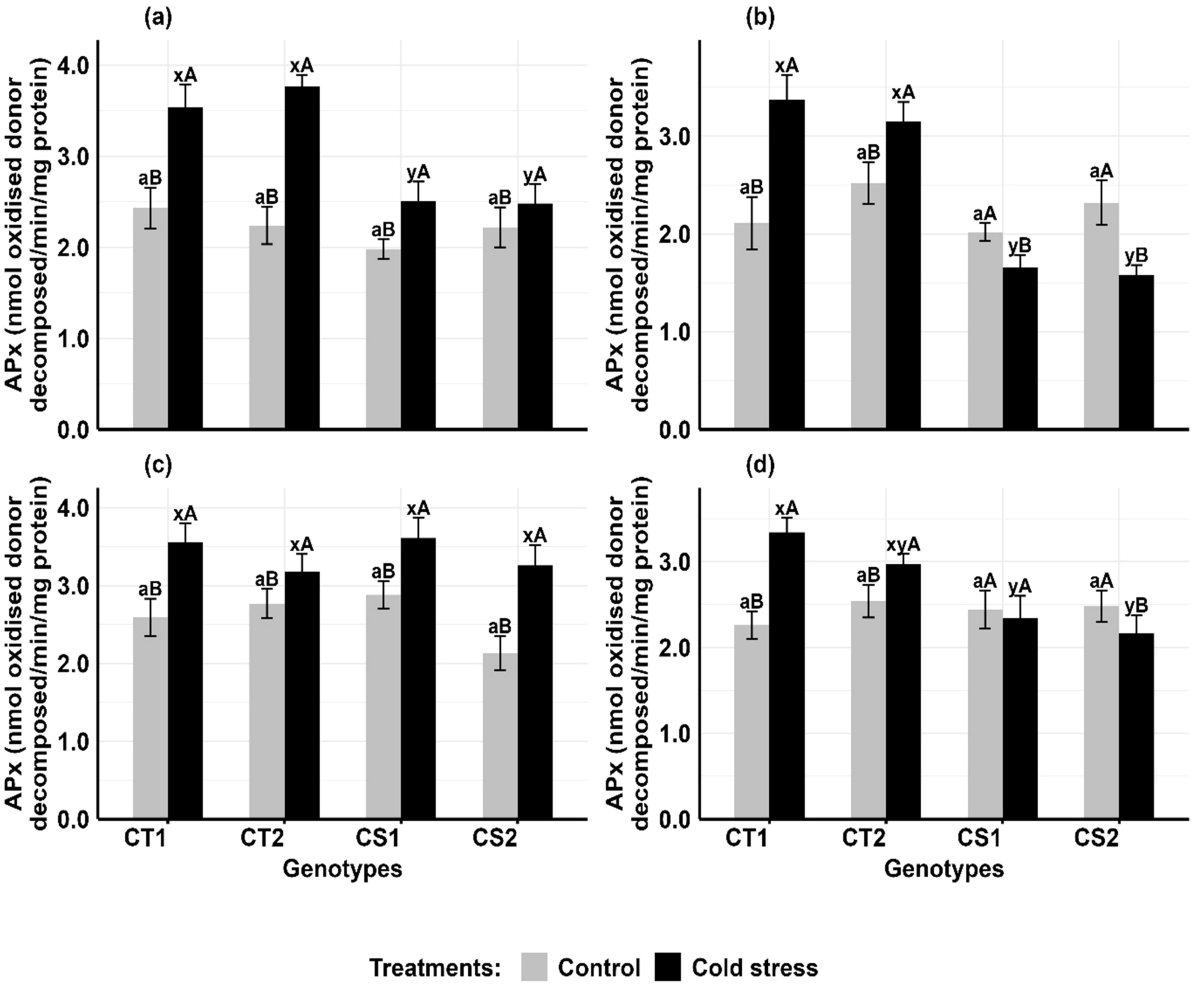
Ascorbate peroxidase (APX) **(a)** Anthers-stage 1, **(b)** Anthers-stage 2, **(c)** Ovules-stage 1, **(d)** Ovules-stage 2, under control and cold stress conditions. CT1: Cold tolerant genotype 1 (ICC 17258); CT2: Cold tolerant genotype 2 (ICC 16349); CS1: Cold sensitive genotype 1 (ICC 15567); CS2: Cold sensitive genotype 2 (GPF-2). Vertical bars represent standard errors (n=3). Genotypic differences within each treatment were analyzed using one-way ANOVA followed by Tukey's HSD test. Different lowercase letters (a for control and x, y for cold stress) denote significant differences (p < 0.05) among genotypes within the same treatment. Treatment differences (Control vs. Cold Stress) within each genotype were analyzed using a paired t-test. Different uppercase letters (A, B) indicate significant differences (p < 0.05) between treatments within the same genotype, where 'A' is indicated for the highest value and 'B' is indicated the smallest value.

Cold stress increased ascorbate peroxidase activity in the anthers and ovules of CT genotypes at both developmental stages, while CS genotypes had variable results (**Figure 5**). In anthers (**Figure 5a, b**), APx activity increased in CT genotypes by 51–80% at stage 1 and 17–39% at stage 2, while CS genotypes increased by 16–28.2% at stage 1 but decreased by 17–27% at stage 2. In ovules (**Figure 5c, d**), APx activity in CT genotypes increased by 34–48% at stage 1 and 15–27% at stage 2, while CS genotypes increased APx activity by 26–40% at stage 1 but decreased by 8–17% at stage 2.

#### 3.5.4 Glutathione reductase

**Figure 6:**
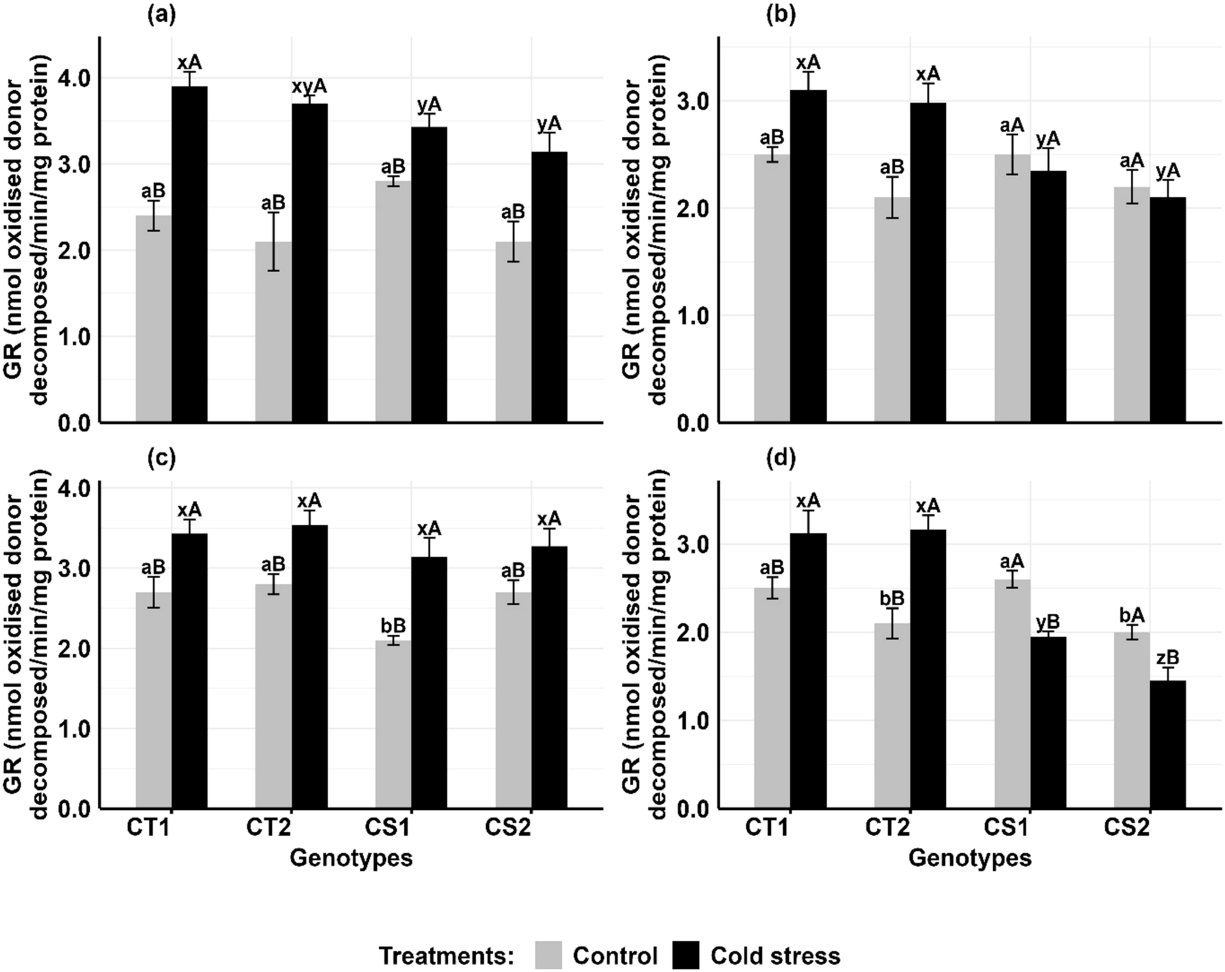
Glutathione reductase (GR) **(a)** Anthers-stage 1, **(b)** Anthers-stage 2, **(c)** Ovules-stage 1, **(d)** Ovules-stage 2, under control and cold stress conditions. CT1: Cold tolerant genotype 1 (ICC 17258); CT2: Cold tolerant genotype 2 (ICC 16349); CS1: Cold sensitive genotype 1 (ICC 15567); CS2: Cold sensitive genotype 2 (GPF-2). Vertical bars represent standard errors (n=3). Genotypic differences within each treatment were analyzed using one-way ANOVA followed by Tukey's HSD test. Different lowercase letters (a, b for control and x, y, z for cold stress) denote significant differences (p < 0.05) among genotypes within the same treatment. Treatment differences (Control vs. Cold Stress) within each genotype were analyzed using a paired t-test. Different uppercase letters (A, B) indicate significant differences (p < 0.05) between treatments within the same genotype, where 'A' is indicated for the highest value and 'B' is indicated the smallest value.

Cold stress increased GR activity in the anthers and ovules of CT genotypes at both developmental stages, while CS genotypes had variable results (**Figure 6**). In anthers (**Figures 6a, b**), GR activity increased in CT genotypes by 62–76% at stage 1 and 24–41% at stage 2, while CS genotypes increased by 22–49% at stage 1 but decreased by 14–16% at stage 2. In ovules (**Figures 6c, d**), GR activity increased in CT genotypes by 26–40% at stage 1 and 24–36% at stage 2, while CS genotypes increased GR activity by 21–36% at stage 1 but decreased by 24–26% at stage 2.

### 3.6 Non-enzymatic antioxidants

#### 3.6.1 Ascorbic acid

**Figure 7:**
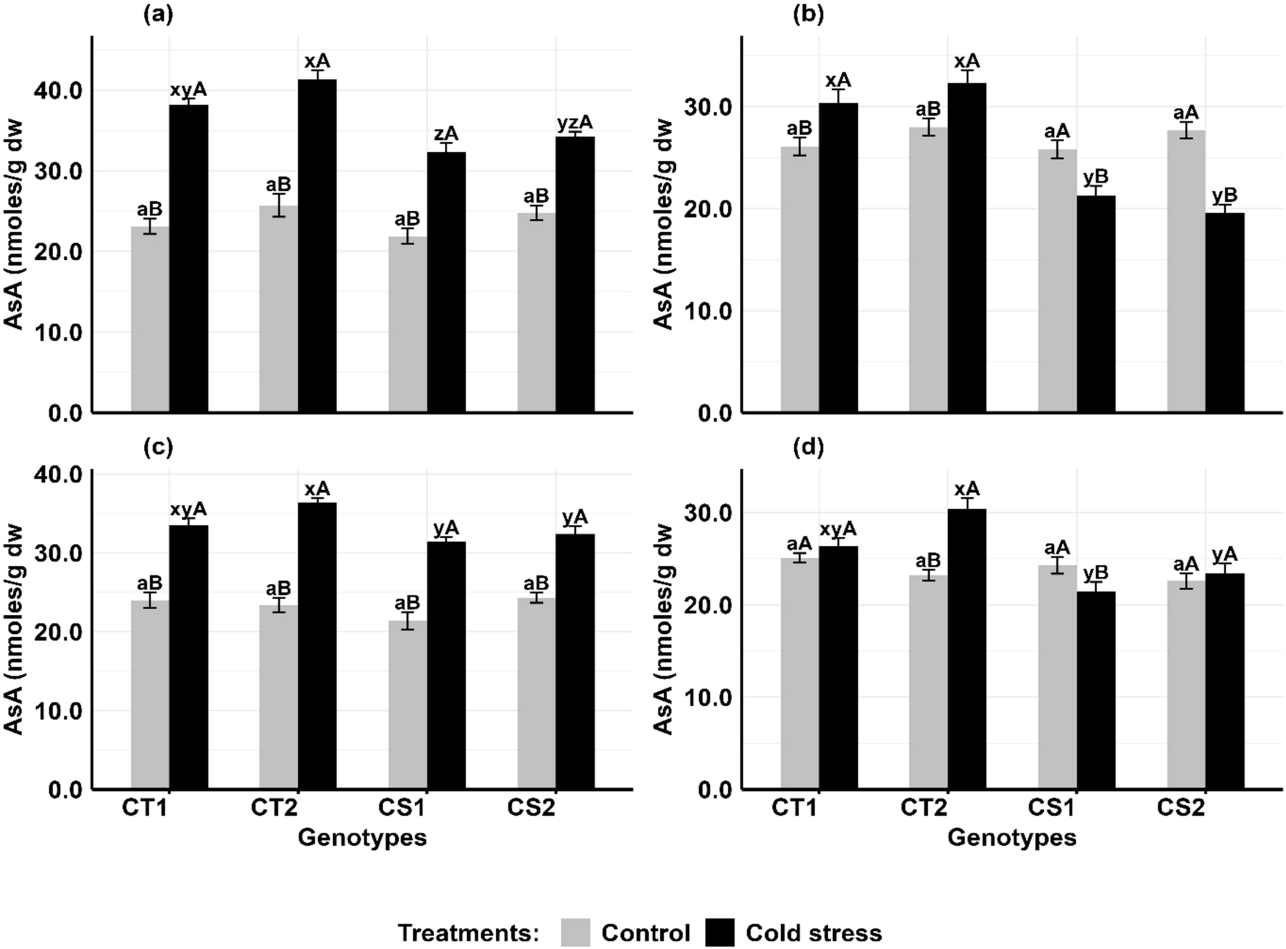
Ascorbic acid (ASA) **(a)** Anthers-stage 1, **(b)** Anthers-stage 2, **(c)** Ovules-stage 1, **(d)** Ovules- stage 2, under control and cold stress conditions. CT1: Cold tolerant genotype 1 (ICC 17258); CT2: Cold tolerant genotype 2 (ICC 16349); CS1: Cold sensitive genotype 1 (ICC 15567); CS2: Cold sensitive genotype 2 (GPF-2). Vertical bars represent standard errors (n=3). Genotypic differences within each treatment were analyzed using one-way ANOVA followed by Tukey's HSD test. Different lowercase letters (a for control and x, y, z for cold stress) denote significant differences (p <0.05) among genotypes within the same treatment. Treatment differences (Control vs. Cold Stress) within each genotype were analyzed using a paired t-test. Different uppercase letters (A, B) indicate significant differences (p < 0.05) between treatments within the same genotype, where 'A' is indicated for the highest value and 'B' is indicated the smallest value.

In anthers (**Figures 7a, b**), ascorbic acid (AsA) levels increased in CT genotypes by 61–65% at stage 1 and 14–16% at stage 2, while CS genotypes increased by 39–49% at stage 1 but declined by 16–29% at stage 2. In ovules (**Figures 7c, d**), AsA levels increased in CT genotypes by 37–55% at stage 1 and 21–31% at stage 2, while CS genotypes increased AsA levels by 33–46% at stage 1 but decreased by 11–14% at stage 2.

#### 3.6.2 Reduced glutathione

**Figure 8:**
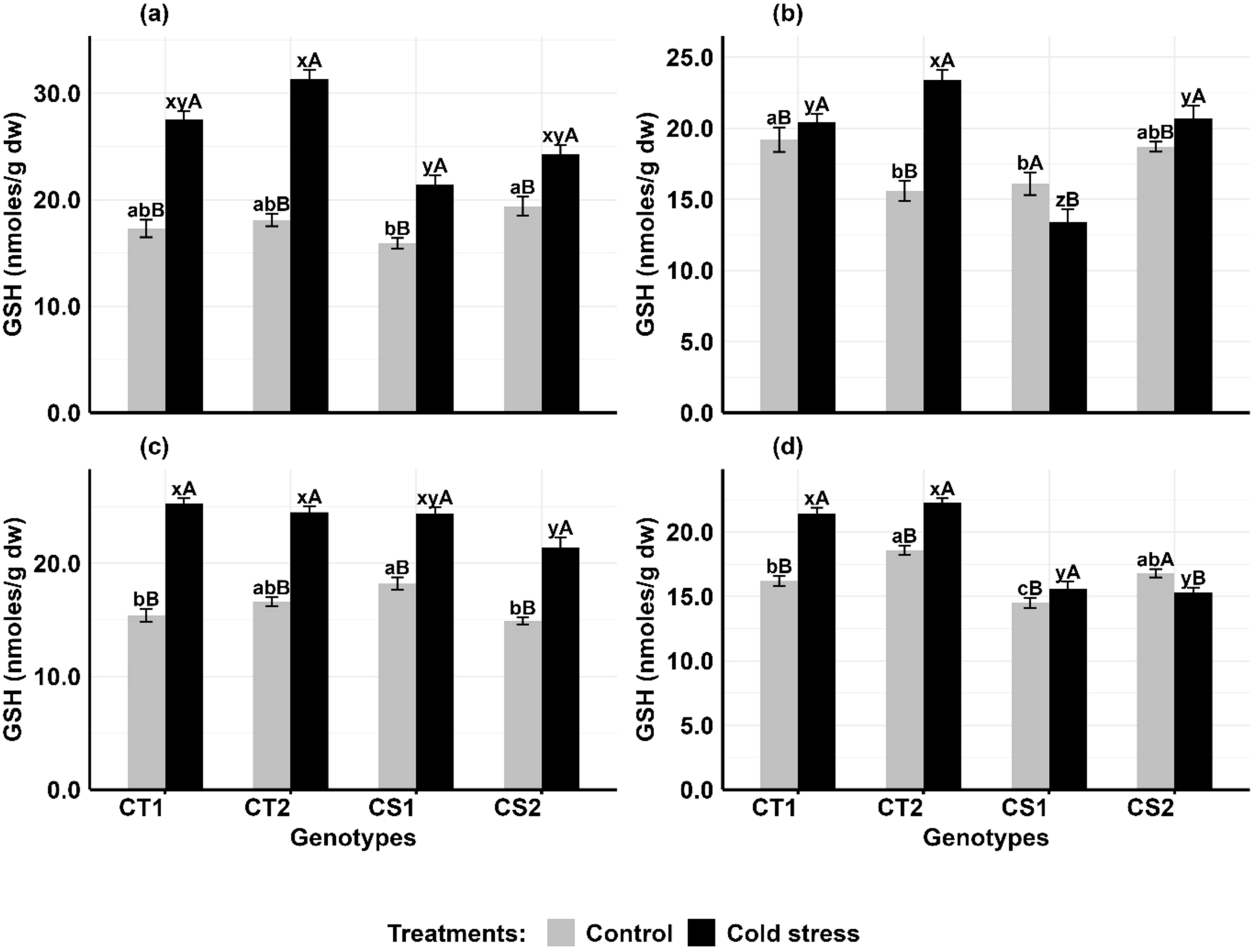
Reduced glutathione (GSH) **(a)** Anthers-stage 1, **(b)** Anthers-stage 2, **(c)** Ovules-stage 1, **(d)** Ovules-stage 2, under control and cold stress conditions. CT1: Cold tolerant genotype 1 (ICC 17258); CT2: Cold tolerant genotype 2 (ICC 16349); CS1: Cold sensitive genotype 1 (ICC 15567); CS2: Cold sensitive genotype 2 (GPF-2). Vertical bars represent standard errors (n=3). Genotypic differences within each treatment were analyzed using one-way ANOVA followed by Tukey's HSD test. Different lowercase letters (a, b, c for control and x, y, z for cold stress) denote significant differences (p<0.05) among genotypes within the same treatment. Treatment differences (Control vs. Cold Stress) within each genotype were analyzed using a paired t-test. Different uppercase letters (A, B) indicate significant differences (p < 0.05) between treatments within the same genotype, where 'A' is indicated for the highest value and 'B' is indicated the smallest value.

In anthers (**Figures 8a, b**), glutathione (GSH) concentrations increased in CT genotypes by 59–72% at stage 1 and 27–38% at stage 2, while CS genotypes increased by 25–34.5% at Stage 1, they exhibited contrasting trends at Stage 2, with one showing an 8% increase and another an 18% decrease. In ovules (**Figures 8c, d**), GSH concentrations in CT genotypes increased by 49–65% at stage 1 and 23–26% at stage 2, while in CS genotypes, it increased by 28–47% at stage 1 but stage 2 showed decreased by 14% and increase of 7%.

### 3.7 Reproductive traits

#### 3.7.1 Pollen viability

**Figure 9:**
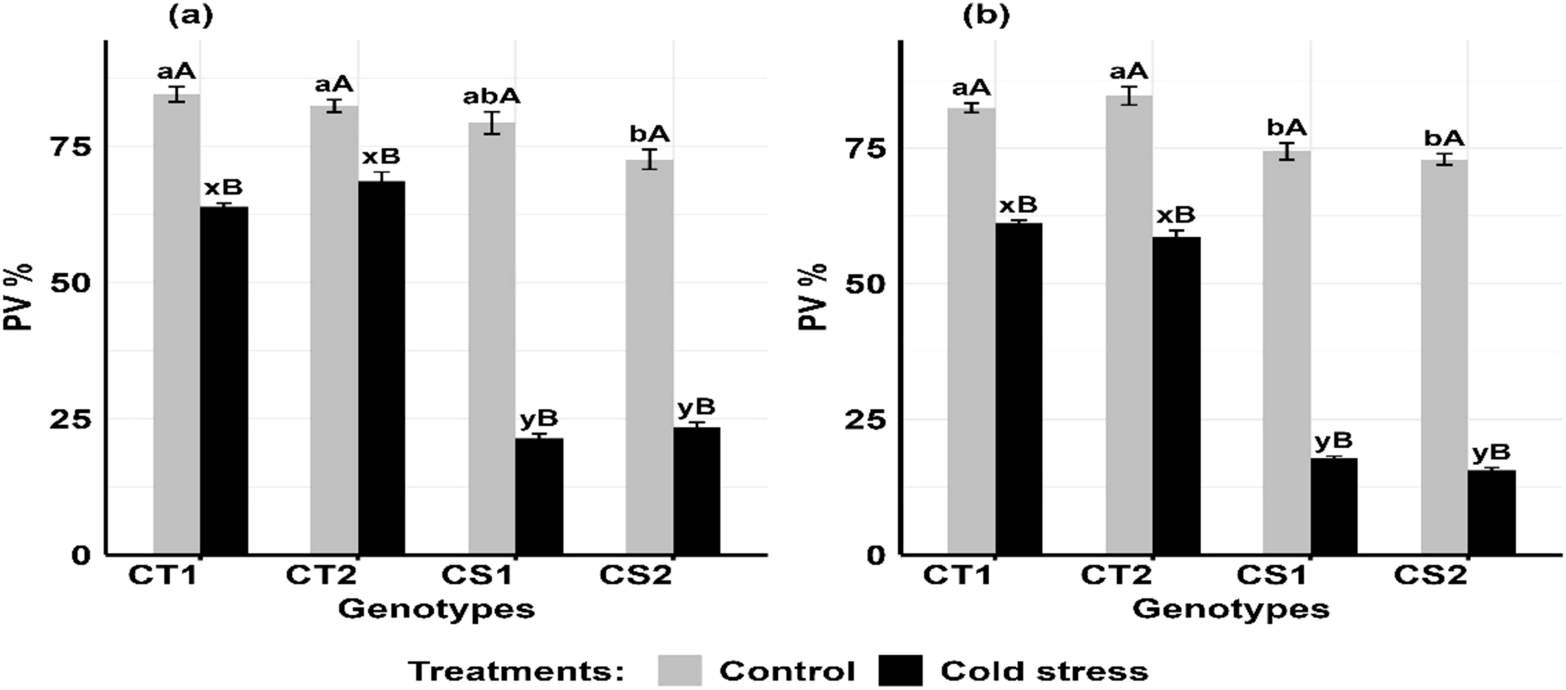
Pollen viability (PV) under control and cold stress conditions at two developmental stages **(a)** stage 1 and **(b)** stage 2. CT1: Cold tolerant genotype 1 (ICC 17258); CT2: Cold tolerant genotype 2 (ICC 16349); CS1: Cold sensitive genotype 1 (ICC 15567); CS2: Cold sensitive genotype 2 (GPF-2). Vertical bars represent standard errors (n=3). Genotypic differences within each treatment were analyzed using one-way ANOVA followed by Tukey's HSD test. Different lowercase letters (a, b for control and x, y for cold stress) denote significant differences (p < 0.05) among genotypes within the same treatment. Treatment differences (Control vs. Cold Stress) within each genotype were analyzed using a paired t-test. Different uppercase letters (A, B) indicate significant differences (p < 0.05) between treatments within the same genotype, where 'A' is indicated for the highest value and 'B' is indicated the smallest value.

Cold stress significantly decreased pollen viability at both developmental stages, particularly in CS genotypes (**Figures 9a, b**). For CT genotypes, pollen viability decreased by 16–22% at stage 1 and 25–32% at stage 2, while in CS genotypes, it declined by 69–73% at stage 1 and 76–78% at stage (**Supplementary Figure S8**).

#### 3.7.2 Pollen germination

**Figure 10:**
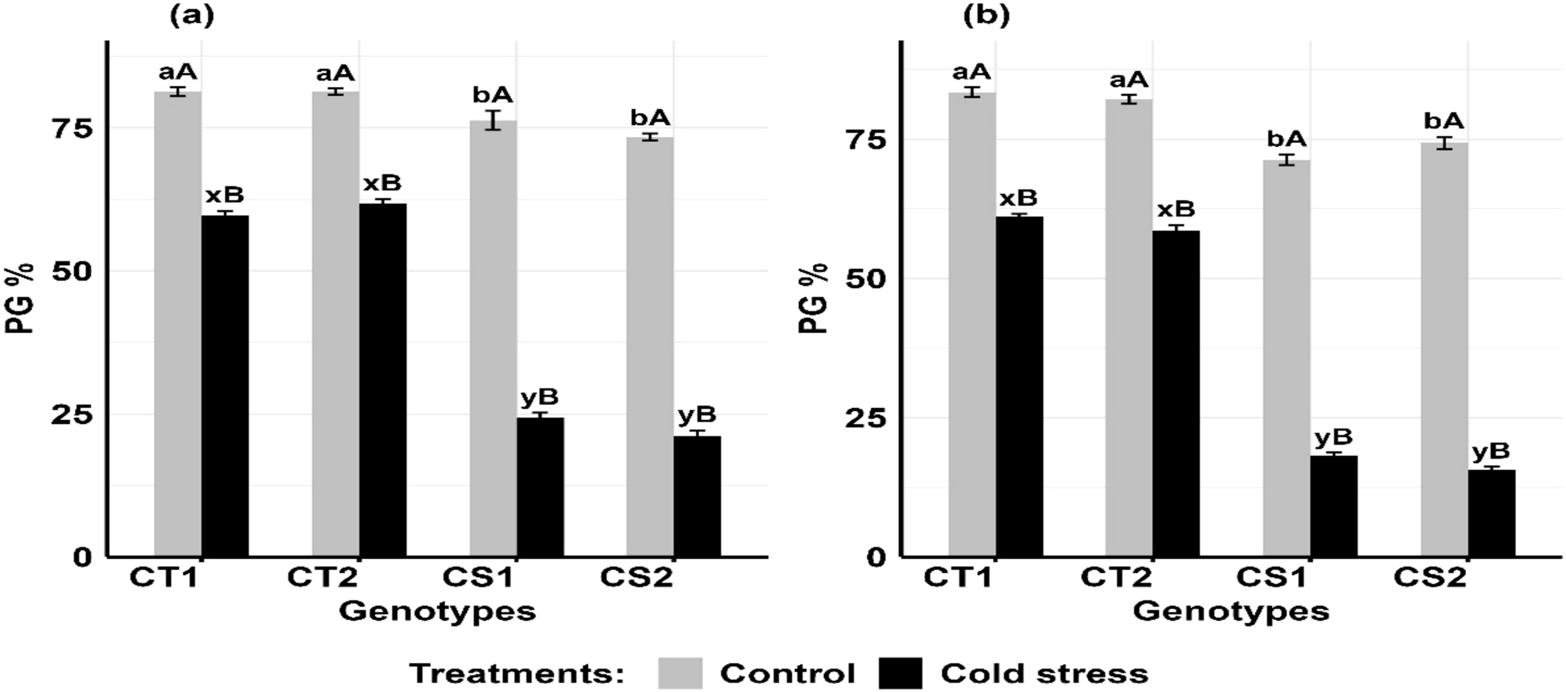
Pollen germination (PG), under control and cold stress conditions at two developmental stages **(a)** stage 1 and **(b)** stage 2. CT1: Cold tolerant genotype 1 (ICC 17258); CT2: Cold tolerant genotype 2 (ICC 16349); CS1: Cold sensitive genotype 1 (ICC 15567); CS2: Cold sensitive genotype 2 (GPF-2). Vertical bars represent standard errors (n=3). Genotypic differences within each treatment were analyzed using one- way ANOVA followed by Tukey's HSD test. Different lowercase letters (a, b for control and x, y for cold stress) denote significant differences (p < 0.05) among genotypes within the same treatment. Treatment differences (Control vs. Cold Stress) within each genotype were analyzed using a paired t-test. Different uppercase letters (A, B) indicate significant differences (p < 0.05) between treatments within the same genotype, where 'A' is indicated for the highest value and 'B' is indicated the smallest value.

Cold stress decreased pollen germination (**Figures 10a, b**) in the CT genotypes by 21–24% at stage 1 and 26–30% at stage 2, while in CS genotypes, it declined by 68–71% at stage 1 and 74–79% at stage 2 (**Supplementary Figure S8**).

#### 3.7.3 Stigma receptivity

**Figure 11:**
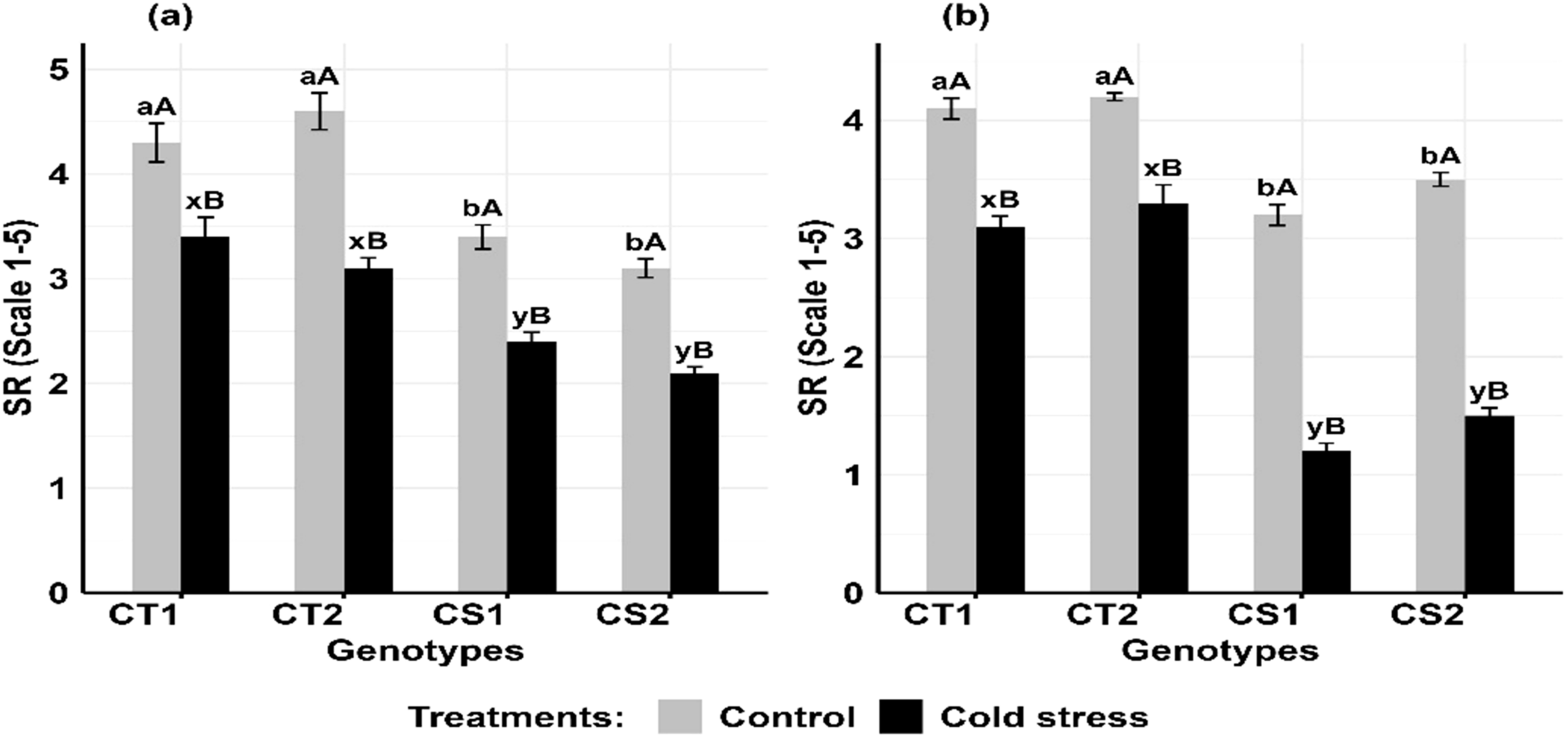
Stigma receptivity (SR), under control and cold stress conditions at two developmental stages **(a)** stage 1 and **(b)** stage 2. CT1: Cold tolerant genotype 1 (ICC 17258); CT2: Cold tolerant genotype 2 (ICC 16349); CS1: Cold sensitive genotype 1 (ICC 15567); CS2: Cold sensitive genotype 2 (GPF-2). Vertical bars represent standard errors (n=3). Genotypic differences within each treatment were analyzed using one-way ANOVA followed by Tukey's HSD test. Different lowercase letters (a, b for control and x, y for cold stress) denote significant differences (p < 0.05) among genotypes within the same treatment. Treatment differences (Control vs. Cold Stress) within each genotype were analyzed using a paired t- test. Different uppercase letters (A, B) indicate significant differences (p < 0.05) between treatments within the same genotype, where 'A' is indicated for the highest and 'B' is for the smallest value.

Cold stress decreased stigma receptivity (**Figures 11a, b**) in the CT genotypes by 11–21% at stage 1 and 14–24% at stage 2, while CS genotypes decreased by 32–33% decrease at stage 1 and 54–59% at stage 2 (**Supplementary Figure S8**).

#### 3.7.4 Ovule viability

**Figure 12:**
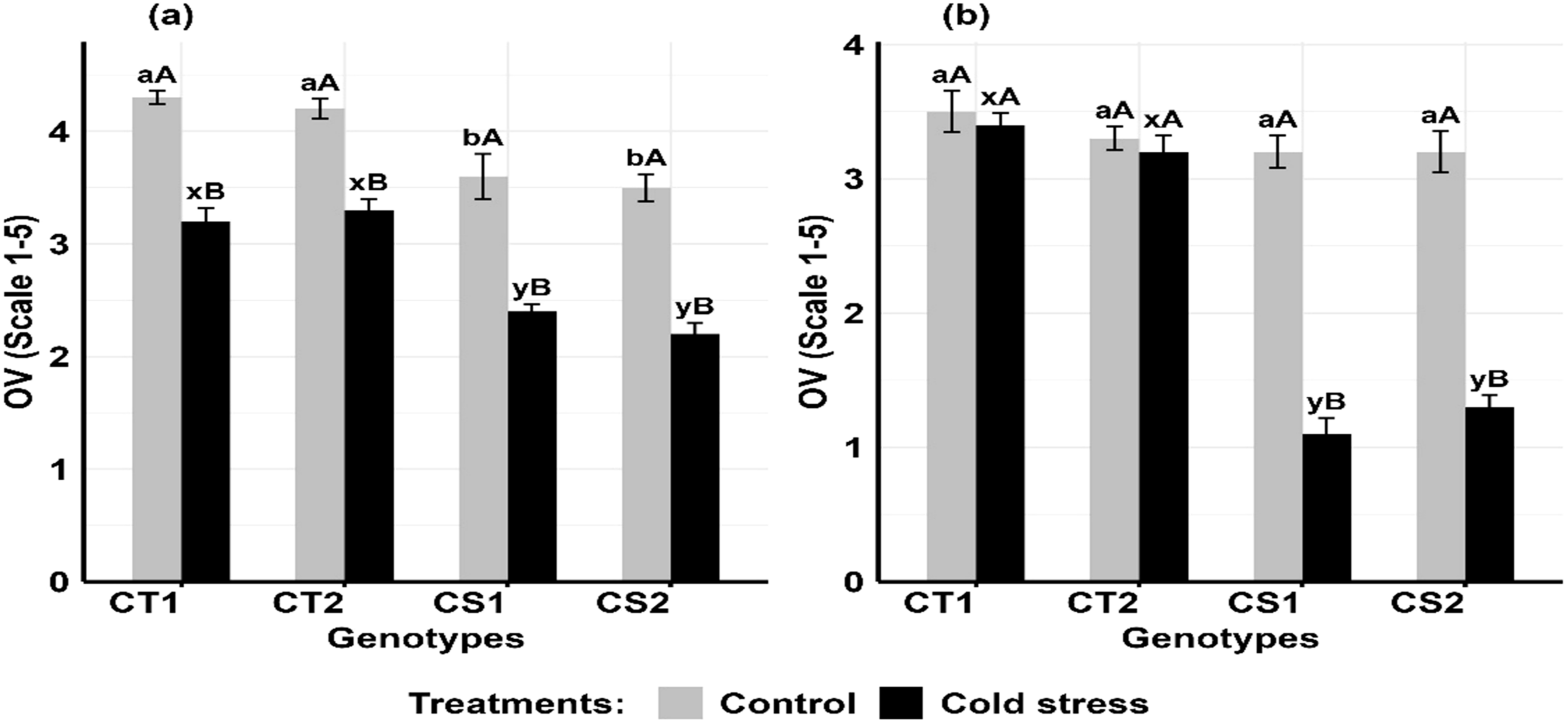
Ovule viability (OV) under control and cold stress conditions at two developmental stages **(a)** stage 1 and **(b)** stage 2. CT1: Cold tolerant genotype 1 (ICC 17258); CT2: Cold tolerant genotype 2 (ICC 16349); CS1: Cold sensitive genotype 1 (ICC 15567); CS2: Cold sensitive genotype 2 (GPF-2). Vertical bars represent standard errors (n=3). Genotypic differences within each treatment were analyzed using one-way ANOVA followed by Tukey's HSD test. Different lowercase letters (a, b for control and x, y for cold stress) denote significant differences (p < 0.05) among genotypes within the same treatment. Treatment differences (Control vs. Cold Stress) within each genotype were analyzed using a paired t- test. Different uppercase letters (A, B) indicate significant differences (p < 0.05) between treatments within the same genotype, where 'A' is indicated for the highest and 'B' is indicated the smallest value.

Cold stress decreased ovule viability (**Figures 12a, b**) in the CT genotypes by 16–19% at stage 1 and 21–27% at stage 2, while CS genotypes decreased it by 31–40% decrease at stage 1 and 59–65% at stage 2 (**Supplementary Figure S8**).

### 3.8 Effects of non-enzymatic antioxidants on pollen function

**Figure 13:**
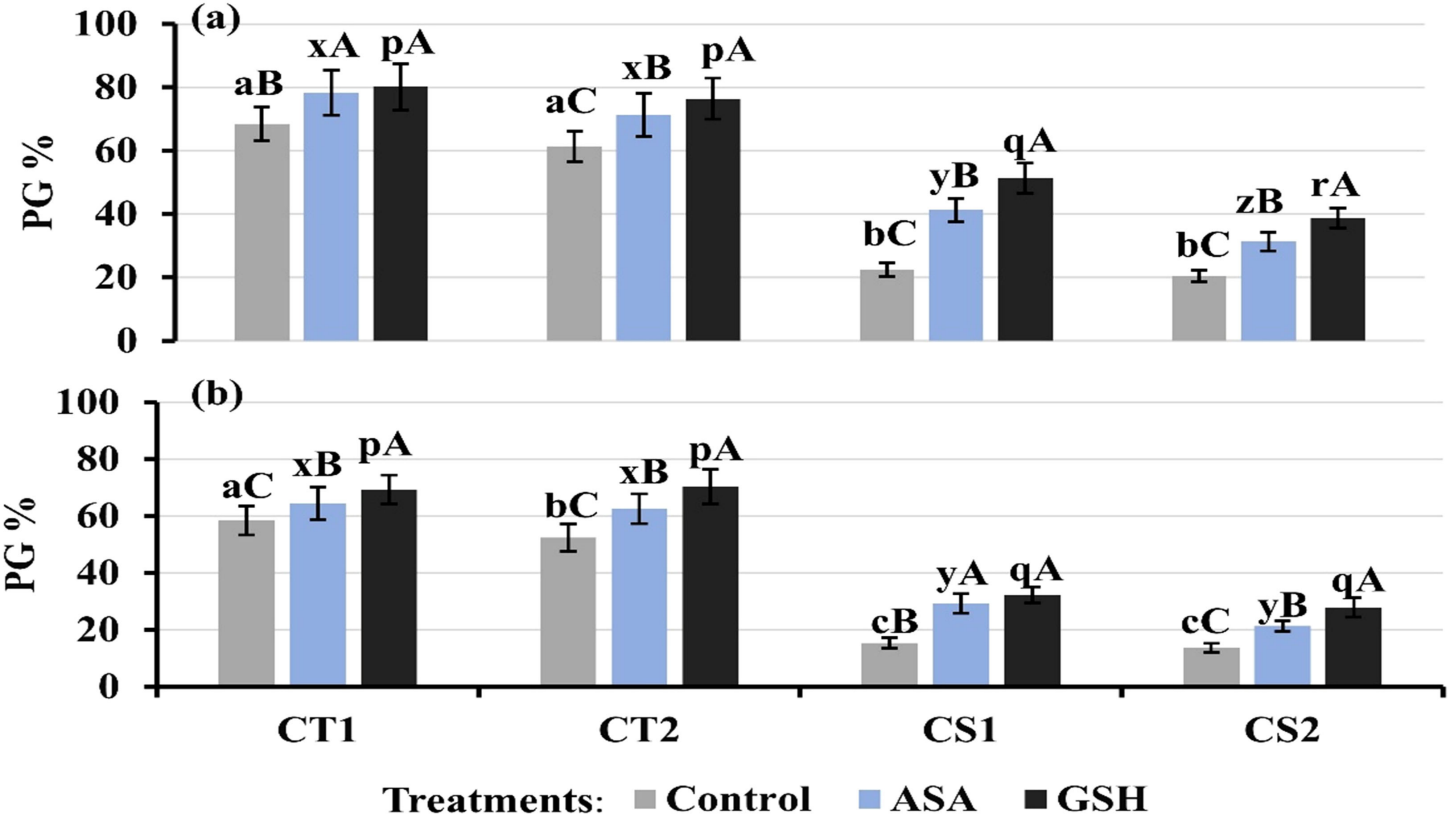
Pollen germination under control conditions and after ascorbic acid (ASA) and reduced glutathione (GSH) treatment at two developmental stages **(a)** Stage 1, **(b)** Stage 2, CT1: Cold tolerant genotype 1 (ICC 17258); CT2: Cold tolerant genotype 2 (ICC 16349); CS1: Cold sensitive genotype 1 (ICC 15567); CS2: Cold sensitive genotype 2 (GPF-2). Vertical bars represent standard errors (n=3). Genotypic differences within each treatment were analyzed using one-way ANOVA followed by Tukey's HSD test. Different lowercase letters (a, b, c for control, x, y, z for AsA, and p, q, r for GSH) denote significant differences (p < 0.05) among genotypes within the same treatment. Treatment differences (Control vs. Cold Stress) within each genotype were analyzed using a paired t-test. Different uppercase letters (A–C) indicate significant differences (p < 0.05) between treatments within the same genotype, where 'A' indicated for the highest value followed by 'B' and 'C' for the smallest value.

Exogenous supplementation with 1 mM ascorbate (AsA) and glutathione (GSH) significantly stimulated pollen germination (**Figures 13a, b**), with pronounced effects in CS genotypes at both developmental stages. Under control (untreated) conditions, pollen germination rates were 61–68% in CT genotypes and 20–22% in CS genotypes at stage 1, and 52–58% in CT genotypes and 13–15% in CS genotypes at stage 2. Under cold stress, the AsA supplementation increased pollen germination rates, especially in CS genotypes, which increased by 31–41% at stage 1 and 21–29% at stage 2. The GSH supplementation resulted in greater pollen germination rates than AsA, particularly in CS genotypes.

### 3.9 Yield traits

**Figure 14:**
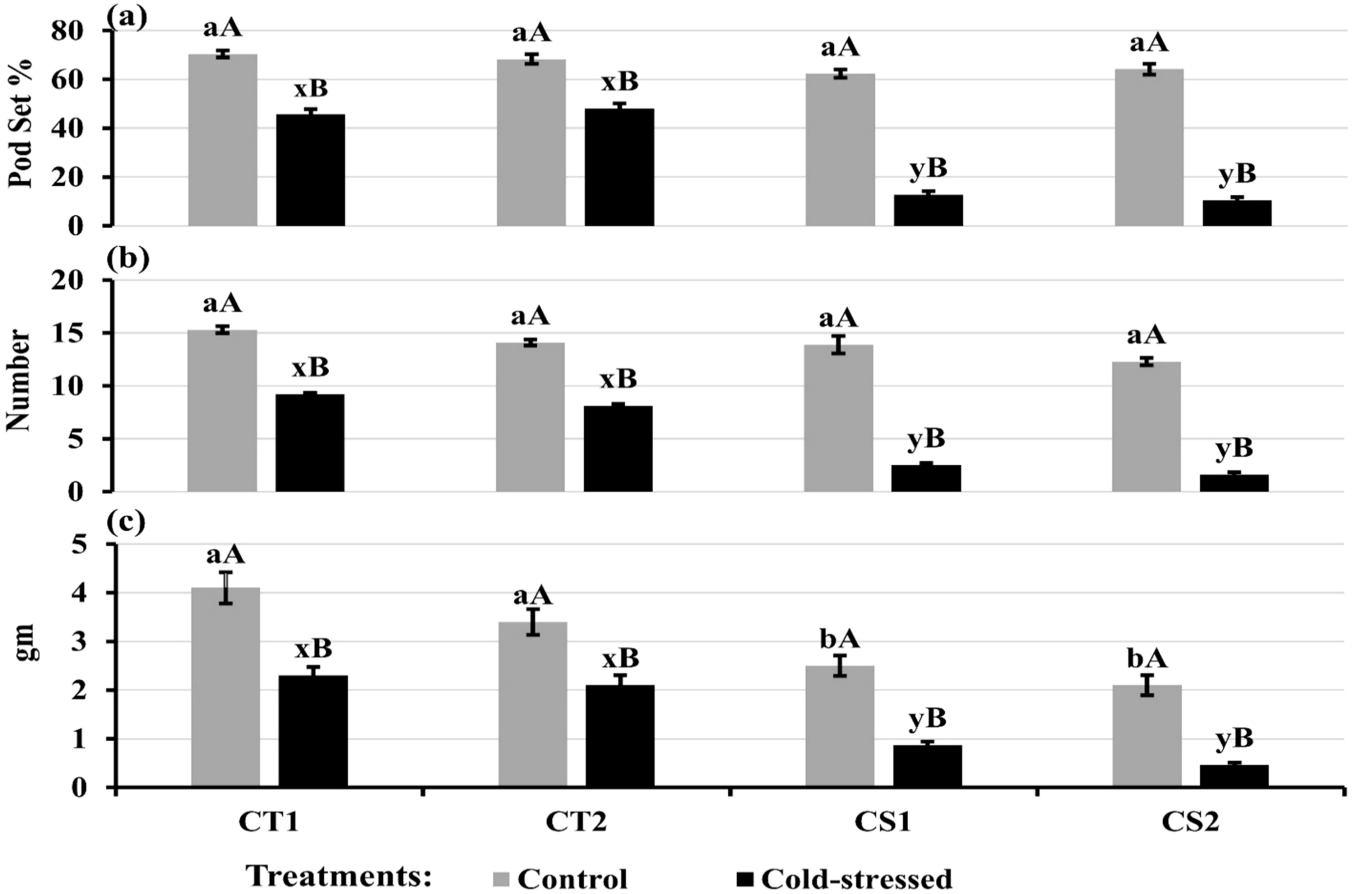
**(a)** Pod set, **(b)** Pod number per plant, and **(c)** Seed weight per plant under control and cold stress conditions. CT1: Cold tolerant genotype 1 (ICC 17258); CT2: Cold tolerant genotype 2 (ICC 16349); CS1: Cold sensitive genotype 1 (ICC 15567); CS2: Cold sensitive genotype 2 (GPF-2). Vertical bars represent standard errors (n=3). Genotypic differences within each treatment were analyzed using one-way ANOVA followed by Tukey's HSD test. Different lowercase letters (a, b for control and x, y for cold stress) denote significant differences (p < 0.05) among genotypes within the same treatment. Treatment differences (Control vs. Cold Stress) within each genotype were analyzed using a paired t- test. Different uppercase letters (A, B) indicate significant differences (p < 0.05) between treatments within the same genotype, where 'A' is indicated for the highest and 'B' is for the smallest value.

Cold stress decreased pod set rates to 45–48% in CT genotypes compared to the control (68–70%) and 10-12% in CS genotypes compared to the control (62–64%) (**Figure 14a**). Moreover, cold stress decreased pod number per plant by 40–42% in CT genotypes and 82–86% in CS genotypes (**Figure 14b**) and seed weight per plant by 38–43% in CT genotypes and 64–79% in CS genotypes (**Figure 14c**).

### 3.10 PCA and Pearson correlation matrix: Anther development stages under cold stress

The PCA **Supplementary Figures S4a**, **S5a**, and **Supplementary Table S2** for another development under cold stress revealed that the first two components (PC1 and PC2) account for 97.79% of the total variation at stage 1 and 98.16% at stage 2. Specifically, PC1 contributed 83.1% of the variance at stage 1 and 92.8% at stage 2, while PC2 contributed 14.7% at stage 1 and 5.39% at stage 2. PC1 positively correlated with variables such as SOD, CAT, APX, AsA, pollen viability, and pollen germination. In contrast, PC1 negatively correlated with EL, MDA, and H_2_O_2_. Conversely, oxidative stress indicators such as MDA and H_2_O_2_ negatively correlated with reproductive traits, which were more pronounced in CS genotypes, and antioxidant enzymes (e.g., SOD and APX) and non-enzymatic antioxidants (e.g., GSH), which were dominant in CT genotypes.

The PCA results also demonstrated that cellular viability had a weak correlation with yield traits at stage 1 but was closer to yield traits at stage 2. The contribution of each variable was represented by a color spectrum ranging from blue (low contribution) to orange (high contribution). Variables such as MDA, H_2_O_2_, EL, PG, and SOD showed significant contributions, marked by intense orange hues.

The Pearson correlation matrix (**Supplementary Figures S4b**, **S5b**) further corroborated these findings, with strong positive correlations among oxidative stress markers (H_2_O_2_, MDA, and EL). Additionally, a strong positive correlation occurred between enzymatic and non-enzymatic antioxidants and between cellular viability and GR activity.

### 3.11 PCA and Pearson correlation matrix: Ovule development stages under cold stress

The PCA analysis (**Supplementary Figures S6a**, **S7a**; **Supplementary Table S3**) for ovule development under cold stress indicated that PC1 and PC2 collectively accounted for a substantial proportion of the variance. At stage 1, PC1 contributed 69.5% of the total variance, while PC2 accounted for 25.9%, together explaining 95.4% of the variance. Similarly, at stage 2, PC1 and PC2 explained 91.47% and 7.08%, respectively, totaling 98.56%. Key variables such as MDA, EL, CAT, APX, and stigma receptivity contributed significantly along PC-1, underscoring their critical role in differentiating genotypes at various developmental stages. A positive association between yield traits and antioxidant activity suggests that enhancing antioxidant defenses can help mitigate oxidative damage, preserving ovule function during critical reproductive stages. Antioxidants such as APX and GR demonstrated stronger associations with yield traits at stage 2, highlighting their increasing importance as the reproductive stage progresses. The contribution levels of variables were represented by a color gradient, with warmer colors (orange and red) indicating higher contributions from variables like MDA, APX, CAT, GSH, and SOD.

The Pearson correlation matrix (**Supplementary Figures S6b**, **S7b**) revealed strong positive correlations between the oxidative stress markers (H_2_O_2_ and MDA) and EL. Conversely, these variables negatively correlated with SOD, CAT, APX, GR, GSH, stigma receptivity, and ovule viability, suggesting opposing responses. Additionally, enzymatic and non-enzymatic antioxidants showed strong positive correlations between floral biology traits such as stigma receptivity and ovule viability.

## 4 Discussion

Cold stress profoundly impacts the reproductive stage of chickpea, leading to significant losses in flowers and pods (Croser et al., 2003; Clarke et al., 2004) due to disruptions in pollen germination, pollen tube growth, fertilization, and pod set (Srinivasan et al., 1999; Kiran et al., 2021; Kaur et al., 2022). While cold-induced damage to reproductive components has been attributed to multiple factors (Nayyar et al., 2005; Rani et al., 2020; Zeitelhofer et al., 2022), few studies have examined oxidative stress as a key contributor. This study systematically evaluated the relative sensitivity of anthers and ovules in chickpea genotypes to cold stress, focusing on oxidative damage at the pre-anthesis and anthesis stages. Employing two cold-tolerant (CT) and two cold-sensitive (CS) genotypes, the research highlights the differential responses of these reproductive structures under prolonged cold stress and provides insight into oxidative damage mechanisms and their correlation with reproductive function.

### 4.1 Impacts of cold stress on cellular function and ROS

Membrane injury, assessed through electrolyte leakage, increased significantly under cold stress in both anthers and ovules, consistent with findings where cold stress compromised membrane structure and functional integrity in chickpea (Mir et al., 2021), wheat (Jan et al., 2023), and rice (Li et al., 2022) through reduced membrane fluidity (Alonso et al., 1997) and increased ion leakage (Campos et al., 2003). Oxidative damage caused by lipid peroxidation exacerbated this damage, as indicated by elevated MDA levels (Chakraborty et al., 2023).

Cold stress significantly impacts cellular viability in chickpea’s reproductive structures, primarily through impaired mitochondrial respiration. Mitochondria are central to energy production, with their functionality critical for maintaining ATP levels, supporting antioxidant defenses, and facilitating metabolic processes that mitigate oxidative damage to reproductive tissues (Rani et al., 2021). Cold stress disrupts mitochondrial function, reducing energy availability and weakening the cell’s ability to counteract oxidative damage (Heidarvand et al., 2017). This decline in cellular viability directly impacts reproductive processes such as pollen tube growth and fertilization, which require adequate energy supply and metabolic stability (De Storme and Geelen, 2014). The relationship between mitochondrial function and oxidative stress is particularly relevant during reproductive development (Karami-Moalem et al., 2018). Excessive ROS accumulation under cold stress can overwhelm antioxidant defenses, leading to oxidative damage to cellular components, including lipids, proteins, and nucleic acids, further compromising cellular viability and reproductive success (Karami-Moalem et al., 2018).

Cold stress increased MDA and H_2_O_2_ levels in the anthers and ovules of CT and CS genotypes. Under cold conditions, ROS accumulation leads to lipid peroxidation (increased MDA), reflecting cellular damage (Morales and Munné-Bosch, 2019). This accumulation of MDA is detrimental to cellular integrity, disrupting membrane structure and function, leading to increased EL and compromised cellular homeostasis in chickpea (Kazemi Shahandashti et al., 2013). Consequently, elevated MDA levels impair vital physiological processes, including reproductive functions, ultimately affecting plant health and yield (Huang et al., 2022). Elevated H_2_O_2_ levels in cold-stressed chickpea plants can disrupt cellular processes by damaging membranes and proteins (Dreyer and Dietz, 2018; Smirnoff and Arnaud, 2019), leading to impaired physiological functions that adversely affect pollen viability and ovule integrity (Nayyar et al., 2005; Hasanuzzaman et al., 2019; Xie et al., 2022). Oxidative damage is particularly detrimental to reproductive structures, as it can hinder fertilization and reduce overall reproductive success, affecting crop yields under cold stress conditions (Soualiou et al., 2022). This study’s observed increase in MDA and H_2_O_2_ levels under cold stress may have significantly impacted membrane damage and cellular viability in chickpea genotypes (de Dios Alché, 2019), adversely affecting cellular function and reproductive structures.

### 4.2 Differential response of anthers and ovules in contrasting chickpea genotypes

This study revealed significant differences in membrane damage (as measured by EL) between reproductive structures under cold stress, with significantly more EL in anthers than ovules, suggesting that the higher metabolic activity associated with pollen production makes them more susceptible to cold-induced damage (Zhang et al., 2020). The CS genotypes experienced more pronounced increases in EL in both reproductive structures, particularly in anthers, indicating heightened physiological stress. In contrast, CT genotypes exhibited less membrane damage, reflecting a more robust physiological response that supports reproductive function under cold stress. These findings are consistent with earlier reports on wheat (Javidi et al., 2022), rice (Rativa et al., 2020), and tomato (Liu et al., 2012), where CT genotypes showed greater membrane stability under cold stress. Increased membrane damage in the anthers and ovules of CS genotypes likely compromises cellular integrity and viability, which are critical for reproductive success. Previous studies have reported significantly better cellular viability in CT genotypes than in CS genotypes in chickpea (Mir et al., 2021; Javidi et al., 2022), rice (Rativa et al., 2020), and potato (Angmo et al., 2023). Anthers showed higher oxidative damage than ovules, as evidenced by elevated ROS levels across developmental stages, especially in CS genotypes. Similar observations have been made in other studies, where cold stress had more severe consequences for male reproductive tissues, resulting in increased pollen abortion rates and reduced fertility compared to female tissues (Zinn et al., 2010; De Storme and Geelen, 2014; Albertos et al., 2019). Excessive oxidative stress can trigger programmed cell death in microspores and ovules, reducing fertility and seed set (De Storme and Geelen, 2014; Huang et al., 2022).

The greater susceptibility of anthers to cold-induced damage compared to ovules may stem from various factors. Anthers are often more exposed than female reproductive structures during critical developmental stages, increasing their vulnerability to environmental stressors, including cold temperatures (Zinn et al., 2010; Hedhly, 2011). Cold stress can disrupt anther meiosis, tapetal development, microsporogenesis, and key physiological processes such as hormone balance and sugar transport, impairing pollen development (Zhang et al., 2022; Liu et al., 2024). This disruption often leads to pollen sterility due to impaired nutrient supply and altered programmed cell death in the tapetum (Huang et al., 2022; Zhang et al., 2021). In contrast, ovules are situated within the ovary, offering some protection from external environmental stress. However, cold stress can still affect ovule development and function (Albertos et al., 2019; Rani et al., 2020). Additionally, differences in developmental stages between male and female components may influence their sensitivity to stress (Hedhly, 2011).

Our findings also revealed that while anthers and ovules possess mechanisms to mitigate oxidative stress under cold conditions, their antioxidant responses differ. Anthers had higher activities of specific enzymes, such as SOD, APX, and GR, along with elevated levels of AsA and GSH, suggesting the crucial role of the ascorbate-glutathione cycle (Foyer and Kunert, 2024). Conversely, ovules exhibited higher CAT activity at both developmental stages. The ascorbate-glutathione (AsA**-**GSH) cycle plays a crucial role in protecting reproductive tissues from oxidative stress, particularly under cold conditions, by maintaining cellular redox homeostasis and detoxifying hydrogen peroxide (H_2_O_2_). This cycle operates in key cellular compartments, including chloroplasts, mitochondria, cytosol, and peroxisomes, where it mitigates oxidative damage caused by excessive reactive oxygen species (ROS). The coordinated action of these enzymes (APX and GR) within the AsA-GSH cycle effectively detoxifies H_2_O_2_ and maintains a high ratio of reduced AsA and GSH, which are critical for protecting cellular components from oxidative damage (Li et al., 2010; Hasanuzzaman et al., 2019). Similar protective mechanisms have been observed in cold-tolerant (CT) genotypes of wheat (Janda et al., 1999) and potato (Angmo et al., 2023), where enhanced AsA-GSH cycle activity was associated with better performance under cold stress.

Correlations between reproductive traits and antioxidant activity showed strong positive associations with APX, GR, AsA, and GSH in anthers and ovules, especially at stage 2, highlighting their importance in sustaining reproductive function. The variation in antioxidant responses reflects the distinct physiological roles of anthers and ovules in reproduction. Anthers maintain pollen viability and fertilization, while ovules concentrate on their development and maturation (Thakur et al., 2010; Zinn et al., 2010). Despite higher antioxidant levels in anthers under stress, ovules can also effectively manage oxidative stress (Ali and Muday, 2024). Both structures have evolved complementary mechanisms to address oxidative challenges, underscoring their interconnected roles in ensuring reproductive success (Ali and Muday, 2024). Exogenous antioxidants such as AsA and GSH effectively reduced the negative impacts of cold stress on pollen germination, especially in the more vulnerable CS genotypes. These results suggest the potential of antioxidant treatments to improve reproductive success in chickpea under adverse conditions. Similar effects have been observed in tomato (Elkelish et al., 2020; Gul et al., 2022), tea (Fu et al., 2023), and bell pepper (Yao et al., 2021), where exogenous applications of AsA and GSH reduced oxidative damage.

Analysis of contrasting genotypes further highlighted that CT genotypes exhibited stronger antioxidant defenses in both anthers and ovules, whereas CS genotypes suffered significant oxidative damage. The superior AsA-GSH cycle efficiency in CT genotypes enables them to maintain pollen viability, prevent premature tapetal cell death, and support ovule integrity under stress, whereas cold-sensitive (CS) genotypes suffer greater oxidative damage due to weaker antioxidant defenses. These findings align with previous studies that reported superior antioxidant mechanisms in CT genotypes of rice (de Freitas et al., 2019), sweet potato (de Araújo et al., 2021), soybean (Hussain et al., 2023), and barley (Valizadeh-Kamran et al., 2018). The enhanced antioxidative capacity of CT genotypes likely reflects evolutionary adaptations to mitigate oxidative stress caused by low temperatures (Mishra et al., 2023).

### 4.3 Variations across developmental stages

Comparing oxidative responses between stage 1 (pre-anthesis) and stage 2 (anthesis) revealed greater oxidative damage at stage 2, despite stage 1 being considered more sensitive to cold stress (Huang et al., 2022). During stage 1, the initial sensitivity to cold triggered cellular damage and oxidative stress responses. However, stage 2 was characterized by cumulative oxidative damage likely due to prolonged cold exposure. As ROS production escalates during this stage, antioxidant defenses in both anthers and ovules become overwhelmed. This depletion of antioxidant reserves (observed in the current study) renders reproductive structures less capable of mitigating oxidative damage at stage 2. Stage 2 coincides with anthesis, a critical period for fertilization and seed set, which makes reproductive structures particularly sensitive to cold stress (Imin et al., 2004; Kiran et al., 2021; Huang et al., 2022). ROS accumulation during anthesis disrupts cellular functions through lipid peroxidation and other damaging processes in anthers and associated components, suggesting that, while stage 1 initiates oxidative stress responses, the inability of CS genotypes to sustain antioxidant defenses into stage 2 is a key factor in the observed oxidative damage. Physiological changes during anthesis, including increased metabolic activity required for pollen tube growth and ovule maturation, further exacerbate the effects of cold stress, generating additional ROS and compounding oxidative stress (Zhou and Dresselhaus, 2023). Previous studies have reported that short-term and long-term exposure to cold stress, which relate to stage 1 and stage 2, respectively in the present study, differentially impact oxidative stress and antioxidant responses in plants. During short-term cold stress, plants experience a rapid increase in reactive oxygen species (ROS) due to the disruption of electron transport chains in the chloroplasts and mitochondria (Gill and Tuteja, 2010). This transient ROS burst can act as a signaling molecule to activate defense mechanisms, including the upregulation of antioxidant enzymes such as superoxide dismutase (SOD), catalase (CAT), and peroxidase (POD) (Prasad et al., 1994). However, if cold stress persists into long-term exposure, the sustained overproduction of ROS overwhelms the antioxidant system, leading to oxidative damage such as lipid peroxidation, protein oxidation, and DNA damage (*Arabidopsis*; Szalai et al., 1996; Sangwan et al., 2002; Chinnusamy et al., 2007). To mitigate this, plants enhance their antioxidant capacity by increasing the activity of enzymes such as ascorbate peroxidase (APX) and glutathione reductase (GR), as well as by accumulating non-enzymatic antioxidants such as ascorbic acid, glutathione, and proline (Kaur and Asthir, 2015). Cold-tolerant species, such as winter wheat and Arabidopsis, exhibit more robust antioxidant responses compared to sensitive species, enabling them to better withstand prolonged cold stress (Janda et al., 1999; Chinnusamy et al., 2007). Thus, while short-term exposure to cold stress primarily triggers signaling pathways, long-term exposure necessitates a sustained antioxidant response to prevent cellular damage and ensure survival. The interplay of these factors emphasizes the importance of understanding the prolonged responses to cold stress. Targeted strategies to enhance antioxidant defenses during anthesis could mitigate the vulnerabilities of chickpea reproductive structures during this critical phase.

The PCA of anthers and ovules under cold stress revealed distinct oxidative stress responses, highlighting their unique roles in reproductive success. Both structures exhibited increased oxidative stress, as indicated by elevated ROS markers (MDA and H_2_O_2_). However, anthers demonstrated a more pronounced negative correlation between yield traits and oxidative stress markers, underscoring the greater vulnerability to oxidative damage, which can severely impair pollen viability. Although ovules also experience oxidative stress, their antioxidant mechanisms appear to confer greater resilience. The PCA results suggest that antioxidants, such as SOD, CAT, APX, and GR, are critical for both anthers and ovules, with the ascorbate-glutathione pathway potentially playing a vital role (see above). In anthers, higher antioxidant activity positively correlated with reproductive traits, highlighting the importance of antioxidants in sustaining pollen development. Conversely, while antioxidants support reproductive success in ovules, their correlation with yield traits appears weaker than in anthers. Notably, the variance explained by the principal components suggests distinct developmental trajectories of anthers and ovules under cold stress, reinforcing the need for stage- and structure-specific approaches to managing oxidative stress in chickpea to ensure reproductive success under cold stress.

## 5 Conclusion

This study demonstrated that anthers and ovules exhibit differential responses to oxidative damage induced by cold stress across the two flower developmental stages. Particularly, anthers displayed greater susceptibility to oxidative damage than ovules, predominantly at anthesis stage. Although both genotypes employed similar reactive oxygen species (ROS) detoxification strategies, the cold-tolerant genotypes demonstrated a superior capacity for ROS management across both the stages, unlike the cold-sensitive genotypes. Among the antioxidants examined, ascorbate peroxidase and glutathione reductase were the most prominent in the cold-tolerant genotypes, accompanied by elevated levels of ascorbate and glutathione in anthers, whereas ovules showed greater expression of catalase. This enhanced ROS regulation improved reproductive function, ultimately leading to enhanced yield traits in the cold-tolerant genotypes. These findings emphasize the importance of elucidating the differential responses of reproductive structures to cold stress to guide the development of cold-resilient chickpea cultivars. Given the observed importance of ascorbate and glutathione in cold-tolerant chickpea genotypes, future studies should evaluate the effectiveness of exogenous application of these antioxidants under realistic field conditions to enhance reproductive cold tolerance. Additionally, further studies should focus on identifying key genes, proteins, and signaling pathways involved in superior ROS management in cold-tolerant genotypes. Understanding these molecular mechanisms could provide targeted strategies for breeding and biotechnological interventions to improve chickpea resilience to cold stress.

## Data Availability

The original contributions presented in the study are included in the article/supplementary material, further inquiries can be directed to the corresponding author/s.
