# Supplementary material for: Differential resilience of chickpea’s reproductive organs to cold stress across developmental stages: insights into antioxidant strategies for enhanced fertility
Source: Front Plant Sci. 2025 Apr 7;16:1545187. doi: 10.3389/fpls.2025.1545187 (PMC12010643; doi:10.3389/fpls.2025.1545187)
Supplement: Supplementary file 1 [file Table1.docx]

**Supplementary Table S1:** ANOVA values for the mean sum of squares for different traits across the four chickpea genotypes

| **Traits** | **Tissue** | **Stages** | **Genotypes** | **Treatment** | **Replicates** | **Genotype*Treatment** | **Error** |
| --- | --- | --- | --- | --- | --- | --- | --- |
| **df** |  |  | 3 | 1 (2) | 2 | 3 (6) | 14 (22) |
| **EL** | Anthers | 1 | 8.93 ** | 241.93 *** | 6.04 * | 11.08 *** | 0.93 |
|  | Anthers | 2 | 35.4 *** | 867.6 *** | 4.2 | 49.2 *** | 1.3 |
|  | Ovules | 1 | 2.92 * | 117.93 *** | 2.17 | 8.25 ** | 0.86 |
|  | Ovules | 2 | 14.5 *** | 342.0 *** | 3.8 *** | 23.6 *** | 0.3 |
| **CV** | Anthers | 1 | 0.0016 | 0.0051 ** | 0.0018 | 0.00007 | 0.0005 |
|  | Anthers | 2 | 0.0021 *** | 0.0222 *** | 0.0075 *** | 0.0014 *** | 0.00006 |
|  | Ovules | 1 | 0.00041 *** | 0.0145 *** | 0.0055 *** | 0.00034 ** | 0.00004 |
|  | Ovules | 2 | 0.0012 *** | 0.016 *** | 0.0034 *** | 0.00023 | 0.000093 |
| **MDA** | Anthers | 1 | 5.4 *** | 444.6 *** | 15.8 *** | 3.6 *** | 0.2 |
|  | Anthers | 2 | 43.1 *** | 1260.1 *** | 15.7 *** | 23.4 *** | 0.1 |
|  | Ovules | 1 | 6.90 *** | 139.20 *** | 11.05 *** | 4.78 *** | 0.09 |
|  | Ovules | 2 | 17.2 *** | 432.7 *** | 5.5 * | 24.8 *** | 1.0 |
| **H_2_O_2_** | Anthers | 1 | 0.250 *** | 4.420 *** | 0.060 * | 0.026 | 0.009 |
|  | Anthers | 2 | 0.360 | 14.821 *** | 0.334 | 0.520 * | 0.110 |
|  | Ovules | 1 | 0.376 *** | 0.626 *** | 0.676 *** | 0.448 *** | 0.030 |
|  | Ovules | 2 | 0.390 ** | 15.12 *** | 0.075 | 0.415 ** | 0.060 |
| **SOD** | Anthers | 1 | 0.79 ** | 2.26 *** | 0.23 | 0.50 * | 0.10 |
|  | Anthers | 2 | 1.23 *** | 0.06 | 0.92 *** | 1.33 *** | 0.032 |
|  | Ovules | 1 | 0.52 *** | 0.56 *** | 0.48 *** | 1.02 *** | 0.02 |
|  | Ovules | 2 | 0.42 *** | 0.088 * | 0.81 *** | 0.612 *** | 0.010 |
| **Cat** | Anthers | 1 | 0.388 *** | 3.527 *** | 0.333 *** | 0.152 ** | 0.021 |
|  | Anthers | 2 | 0.2520 * | 0.065 | 0.149 | 0.828 *** | 0.055 |
|  | Ovules | 1 | 0.598 *** | 8.132 *** | 0.245 ** | 0.554 *** | 0.037 |
|  | Ovules | 2 | 0.879 *** | 0.949 *** | 0.2633 *** | 0.606 *** | 0.018 |
| **APx** | Anthers | 1 | 0.993 *** | 4.412 *** | 0.361 * | 0.482 ** | 0.081 |
|  | Anthers | 2 | 1.732 *** | 0.155 * | 0.790 *** | 1.103 *** | 0.017 |
|  | Ovules | 1 | 0.410 *** | 5.387 *** | 1.179 *** | 0.040 * | 0.011 |
|  | Ovules | 2 | 0.356 *** | 0.453 *** | 0.944 *** | 0.574 *** | 0.011 |
| **GR** | Anthers | 1 | 0.195 | 6.966 *** | 0.101 | 0.247 | 0.121 |
|  | Anthers | 2 | 0.469 *** | 0.604 *** | 0.646 *** | 0.238 *** | 0.0081 |
|  | Ovules | 1 | 0.206 * | 4.576 *** | 0.186 | 0.096 | 0.060 |
|  | Ovules | 2 | 1.509 *** | 0.102 * | 0.452 *** | 1.080 *** | 0.0127 |
| **AsA** | Anthers | 1 | 41.9 *** | 960.1 *** | 9.6 * | 13.8 ** | 1.8 |
|  | Anthers | 2 | 63.89 *** | 6.10 | 4.23 | 56.01 *** | 2.72 |
|  | Ovules | 1 | 13.9 *** | 620.2 *** | 31.8 *** | 5.3 *** | 0.2 |
|  | Ovules | 2 | 22.27 | 9.50 | 17.313 | 26.69 | 0.25 |
| **GSH** | Anthers | 1 | 37.6 *** | 421.7 *** | 14.1 *** | 18.3 *** | 0.2 |
|  | Anthers | 2 | 40.00 *** | 21.28 *** | 12.05 *** | 29.44 *** | 0.18 |
|  | Ovules | 1 | 10.5 *** | 364.3 *** | 6.1 *** | 5.1 *** | 0.2 |
|  | Ovules | 2 | 39.47 *** | 30.15 *** | 1.43 * | 14.18 *** | 0.36 |
| **PV** | | 1 | 1340 *** | 7371 *** | 15 | 679 *** | 5 |
|  |  | 2 | 1334 *** | 10086 *** | 5 | 518 *** | 3 |
| **PG** | | 1 | 1008 *** | 7754 *** | 3 | 462 *** | 3 |
|  |  | 2 | 1370 *** | 9306 *** | 0 | 513 *** | 2 |
| **SR** | | 1 | 2.58 *** | 7.70 *** | 0.112 | 0.034 | 0.045 |
|  |  | 2 | 3.57 *** | 14.57 *** | 0.026 | 0.469 *** | 0.022 |
| **OV** | | 1 | 1.789 *** | 6.10 *** | 0.061 | 0.054 | 0.036 |
|  |  | 2 | 2.284 *** | 7.260 *** | 0.045 | 1.762 *** | 0.042 |
| **PG*** | | 1 | 4493*** | 1115*** | 4 | 40** | 10 |
|  |  | 2 | 4754*** | 679*** | 2 | 12 | 7 |
| **PS** | | | 829.7 *** | 8273.3 *** | 34.6 * | 441.7 *** | 5.4 |
| **PN** | | | 52.006 *** | 315.37 *** | 0.782 | 1.566 * | 0.382 |
| **SW** | | | 3.564 *** | 19.08 *** | 0.486 * | 0.390 * | 0.075 |

Abbreviation: EL Electrolyte leakage, CV Cellular viability, MDA malondialdehyde, H**_2_**O**_2_** hydrogen peroxide, SOD superoxide dismutase, Cat Catalase, APx Ascorbate peroxidase, GR glutathione reductase, AsA ascorbic acid, GSH reduced glutathione, PV pollen viability, PG pollen germination, SR stigma receptivity, OV ovule viability, PS pod set, PN pod number per plant, SW seed weight per plant

Values in the bracket for PG* pollen germination (exogenous treatment)

Significance. codes: - ‘***’ 0.001, ‘**’ 0.01, ‘*’ 0.05, “” ns

**Supplementary Table S2:** Factor analysis, eigenvalue, total variance, and cumulative variance percentage for the principal component analysis (PCA) of both stages of anther development

| **Variables** | **Anther stage 1** | | **Anther stage 2** | |
| --- | --- | --- | --- | --- |
|  | **PC1** | **PC2** | **PC1** | **PC2** |
| **EL** | -0.272 | 0.375 | -0.297 | 0.055 |
| **CV** | 0.159 | 0.607 | 0.284 | -0.146 |
| **MDA** | -0.270 | 0.379 | -0.297 | 0.149 |
| **H_2_O_2_** | -0.311 | 0.052 | -0.295 | -0.033 |
| **SOD** | 0.315 | 0.004 | 0.299 | -0.006 |
| **CAT** | 0.285 | 0.253 | 0.292 | 0.011 |
| **APX** | 0.316 | 0.019 | 0.297 | -0.029 |
| **GR** | 0.278 | -0.357 | 0.293 | -0.233 |
| **AsA** | 0.300 | 0.232 | 0.298 | -0.035 |
| **GSH** | 0.287 | 0.306 | 0.194 | 0.946 |
| **PV** | 0.314 | 0.009 | 0.298 | 0.007 |
| **PG** | 0.315 | -0.048 | 0.298 | -0.007 |
| **Eigenvalue** | 9.97 | 1.76 | 11.1 | 0.64 |
| **Total variance %** | 83.09 | 14.66 | 92.7 | 5.39 |
| **Cumulative %** | 83.09 | 97.76 | 92.7 | 98.16 |

Abbreviations: EL electrolyte leakage, CV cellular viability, MDA malondialdehyde content, H_2_O_2_ hydrogen peroxide, SOD superoxide dismutase, CAT catalase, APX ascorbate peroxidase, GR glutathione reductase, AsA ascorbic acid, GSH reduced glutathione, PV pollen viability and PG pollen germination.

**Supplementary Table S3:** Factor analysis, eigenvalue, total variance, and cumulative variance percentage for the principal component analysis (PCA) of both stages of ovule development.

| **Variables** | **Ovules Stage 1** | | **Ovules Stage 2** | |
| --- | --- | --- | --- | --- |
|  | **PC1** | **PC2** | **PC1** | **PC2** |
| **EL** | -0.323 | -0.010 | -0.296 | 0.183 |
| **CV** | 0.125 | 0.511 | 0.209 | 0.764 |
| **MDA** | -0.277 | 0.338 | -0.296 | 0.192 |
| **H_2_O_2_** | -0.322 | 0.157 | -0.295 | 0.216 |
| **SOD** | 0.340 | 0.051 | 0.298 | -0.050 |
| **CAT** | 0.331 | 0.161 | 0.300 | -0.075 |
| **APX** | -0.036 | -0.564 | 0.291 | -0.161 |
| **GR** | 0.311 | 0.247 | 0.295 | -0.200 |
| **AsA** | 0.275 | 0.282 | 0.271 | 0.427 |
| **GSH** | 0.304 | -0.265 | 0.300 | -0.048 |
| **OV** | 0.321 | -0.168 | 0.296 | 0.194 |
| **SR** | 0.320 | -0.107 | 0.298 | 0.001 |
| **Eigenvalue** | 8.34 | 3.10 | 10.97 | 0.85 |
| **Total variance %** | 69.53 | 25.86 | 91.47 | 7.08 |
| **Cumulative %** | 69.53 | 95.40 | 91.47 | 98.56 |

Abbreviations: EL electrolyte leakage, CV cellular viability, MDA malondialdehyde content, H_2_O_2_ hydrogen peroxide, SOD superoxide dismutase, CAT catalase, APX ascorbate peroxidase, GR glutathione reductase, AsA ascorbic acid, GSH reduced glutathione, OV ovule viability and SR stigma receptivity.
