# Supplementary material for: Differential resilience of chickpea’s reproductive organs to cold stress across developmental stages: insights into antioxidant strategies for enhanced fertility
Source: Front Plant Sci. 2025 Apr 7;16:1545187. doi: 10.3389/fpls.2025.1545187 (PMC12010643; doi:10.3389/fpls.2025.1545187)
Supplement: Supplementary file 2 [file Table2.docx]

**Supplementary Fig. S1:** Temperature (℃) data (Max: maximum; Min: minimum; Avg: average) recorded in the outdoor environment from sowing to 50 days after sowing


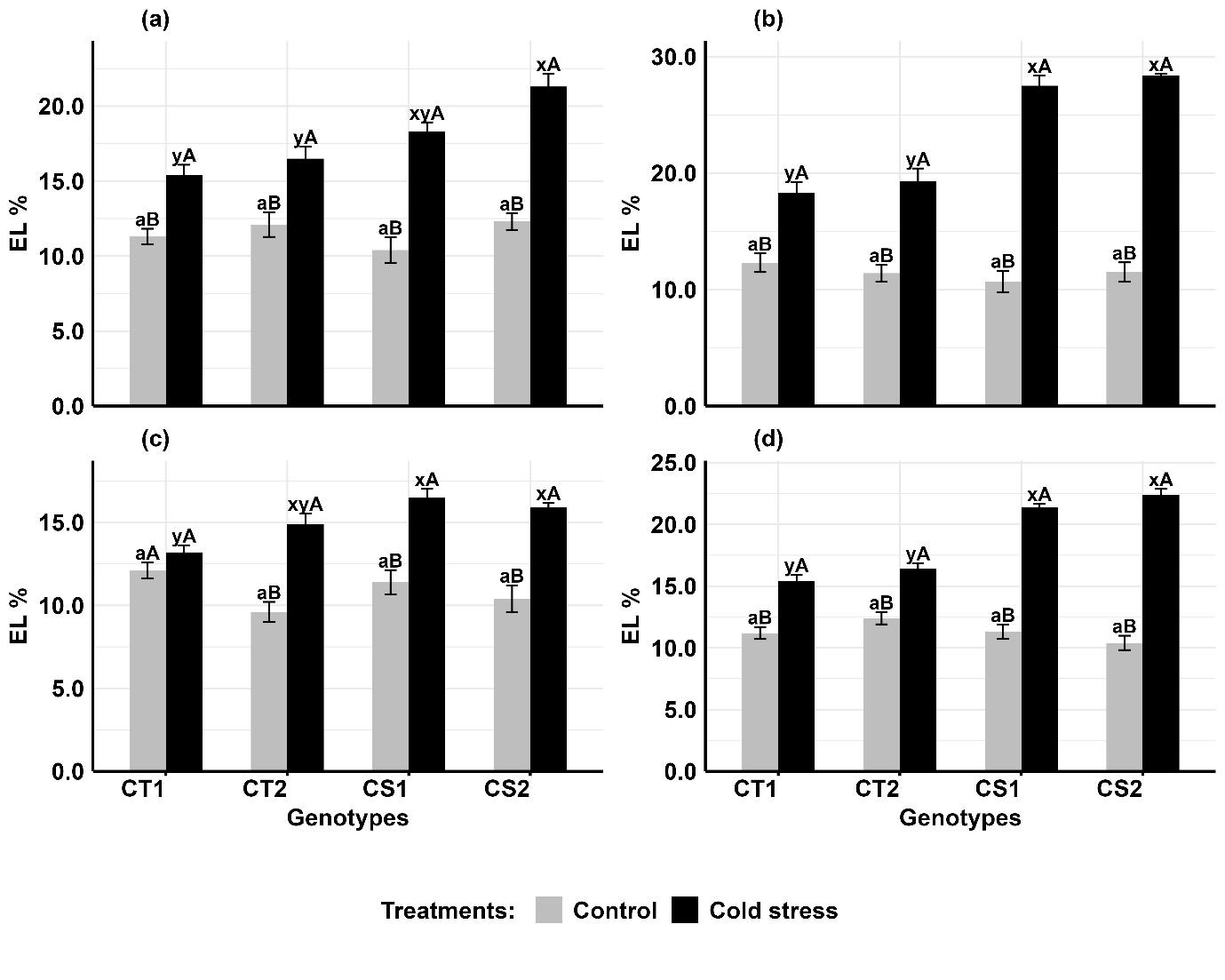


**Supplementary Fig. S2:** Electrolyte leakage (EL): a) Anthers-stage 1, b) Anthers-stage 2, c) Ovules-stage 1, d) Ovules-stage 2, under control and cold stress conditions. CT1: Cold tolerant genotype 1 (ICC 17258); CT2: Cold tolerant genotype 2 (ICC 16349); CS1: Cold sensitive genotype 1 (ICC 15567); CS2: Cold sensitive genotype 2 (GPF-2). Vertical bars represent standard errors (n=3). Genotypic differences within each treatment were analyzed using **one-way ANOVA followed by Tukey’s HSD test**. Different lowercase letters (a for control and x, y for cold stress) denote significant differences (p < 0.05) among genotypes within the same treatment. Treatment differences (Control vs. Cold Stress) within each genotype were analyzed using **a paired t-test**. Different **uppercase letters** (A, B) indicate significant differences (p < 0.05) between treatments within the same genotype, where 'A' is indicated for the highest and 'B' is for the smallest value.

**
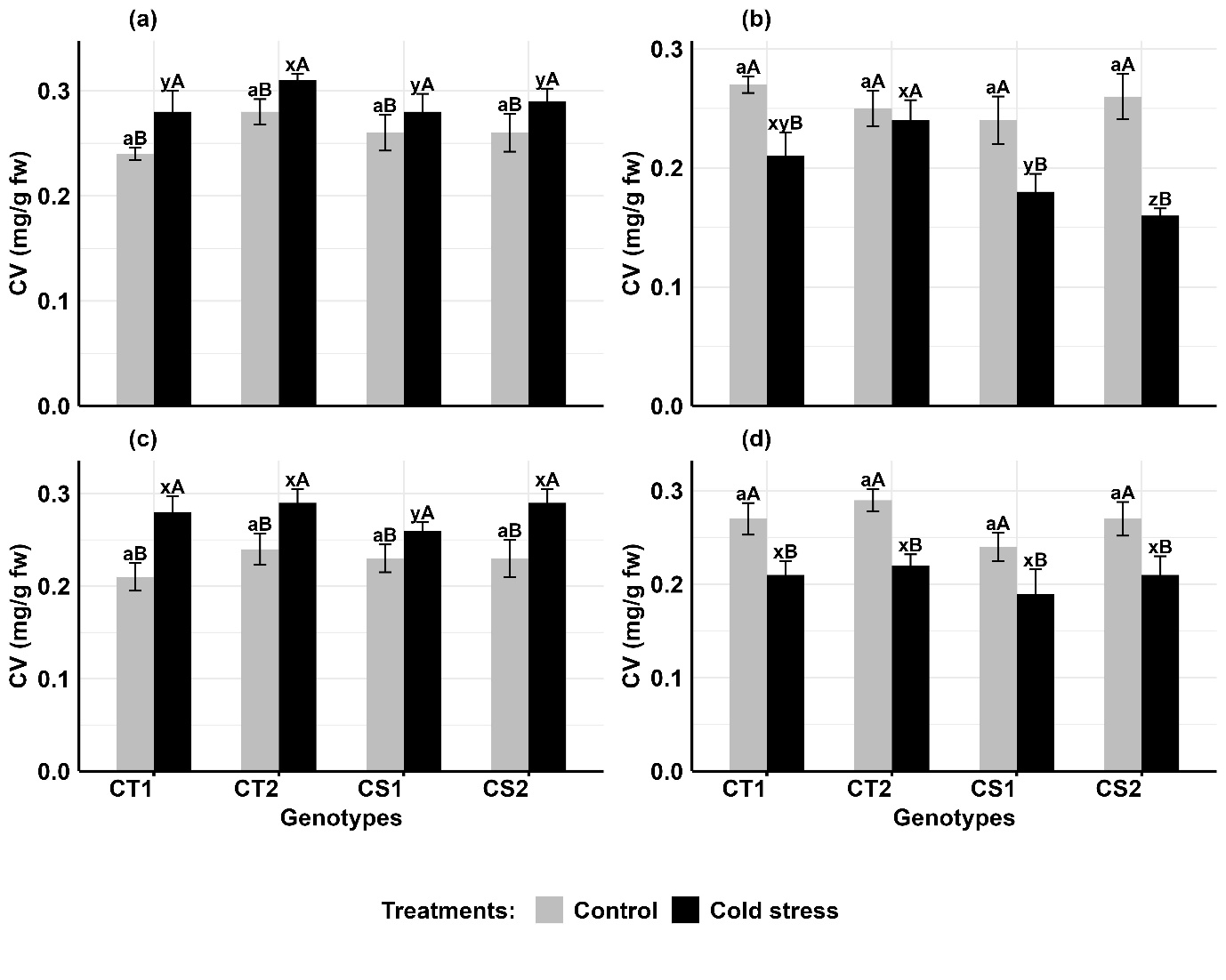
**

**Supplementary Fig. S3:** Cellular viability (CV) a) Anthers-stage 1, b) Anthers-stage 2, c) Ovules-stage 1, d) Ovules-stage 2, under control and cold stress conditions. CT1: Cold tolerant genotype 1 (ICC 17258); CT2: Cold tolerant genotype 2 (ICC 16349); CS1: Cold sensitive genotype 1 (ICC 15567); CS2: Cold sensitive genotype 2 (GPF-2). Vertical bars represent standard errors (n=3). Genotypic differences within each treatment were analyzed using **one-way ANOVA followed by Tukey’s HSD test**. Different lowercase letters (a for control and x, y, z for cold stress) denote significant differences (p < 0.05) among genotypes within the same treatment. Treatment differences (Control vs. Cold Stress) within each genotype were analyzed using **a paired t-test**. Different **uppercase letters** (A, B) indicate significant differences (p < 0.05) between treatments within the same genotype, where 'A' is indicated for the highest and 'B' is for the smallest value.


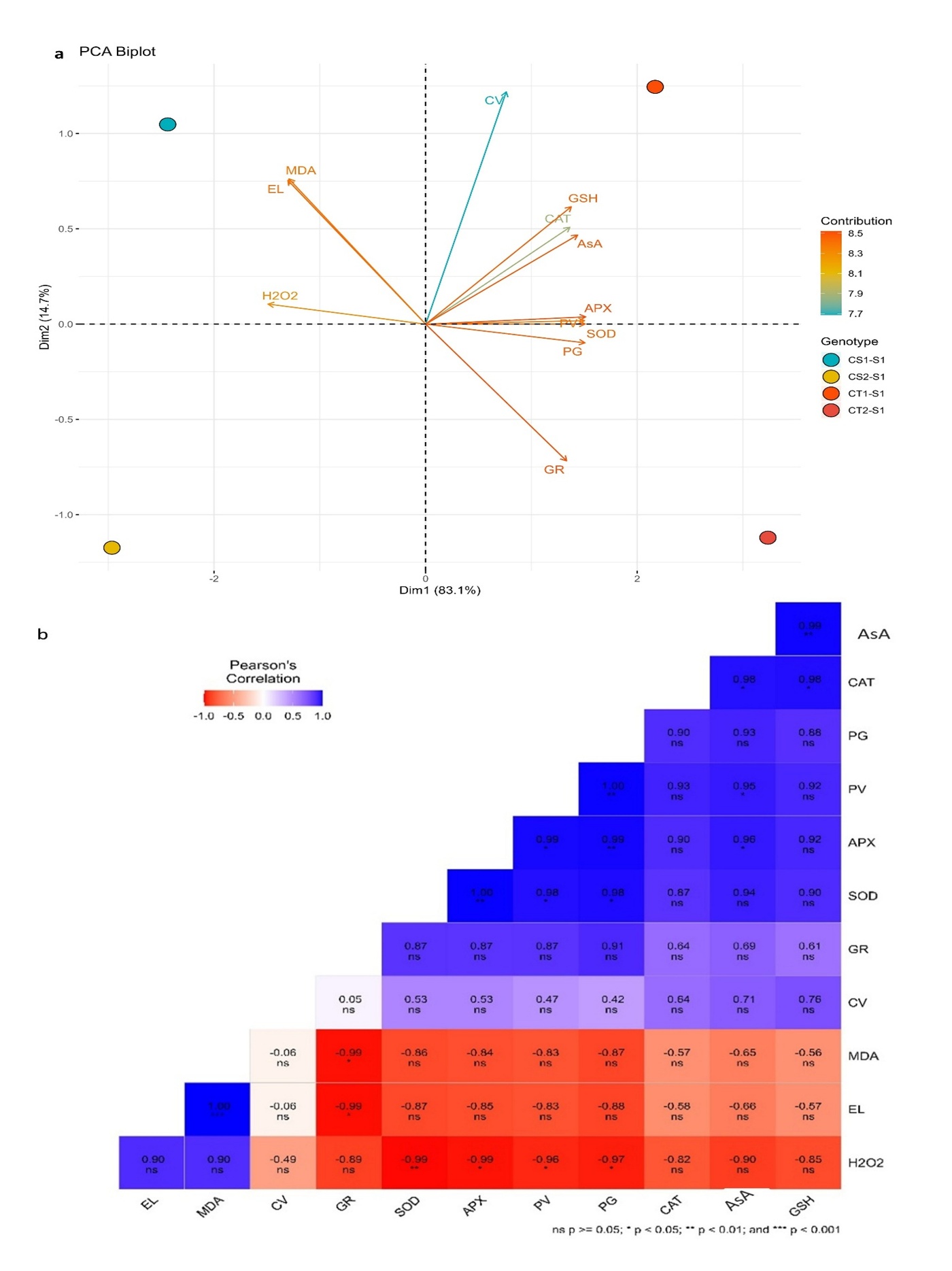


**Supplementary Fig. S4:** (a) Principal component analysis (PCA) biplot for 12 variables under cold stress at stage 1 (pre-anthesis) of anther developmental, and (b) Pearson correlation matrix. EL: electrolyte leakage; CV: cellular viability; MDA: malondialdehyde content; H_2_O_2_: hydrogen peroxide; SOD: superoxide dismutase; CAT: catalase; APX: ascorbate peroxidase; GR: glutathione reductase; AsA: ascorbic acid; GSH: reduced glutathione; PV: pollen viability; PG: pollen germination. CT1: Cold tolerant genotype 1 (ICC 17258); CT2: Cold tolerant genotype 2 (ICC 16349); CS1: Cold sensitive genotype 1 (ICC 15567); CS2: Cold sensitive genotype 2 (GPF-2). S1-Stage 1.


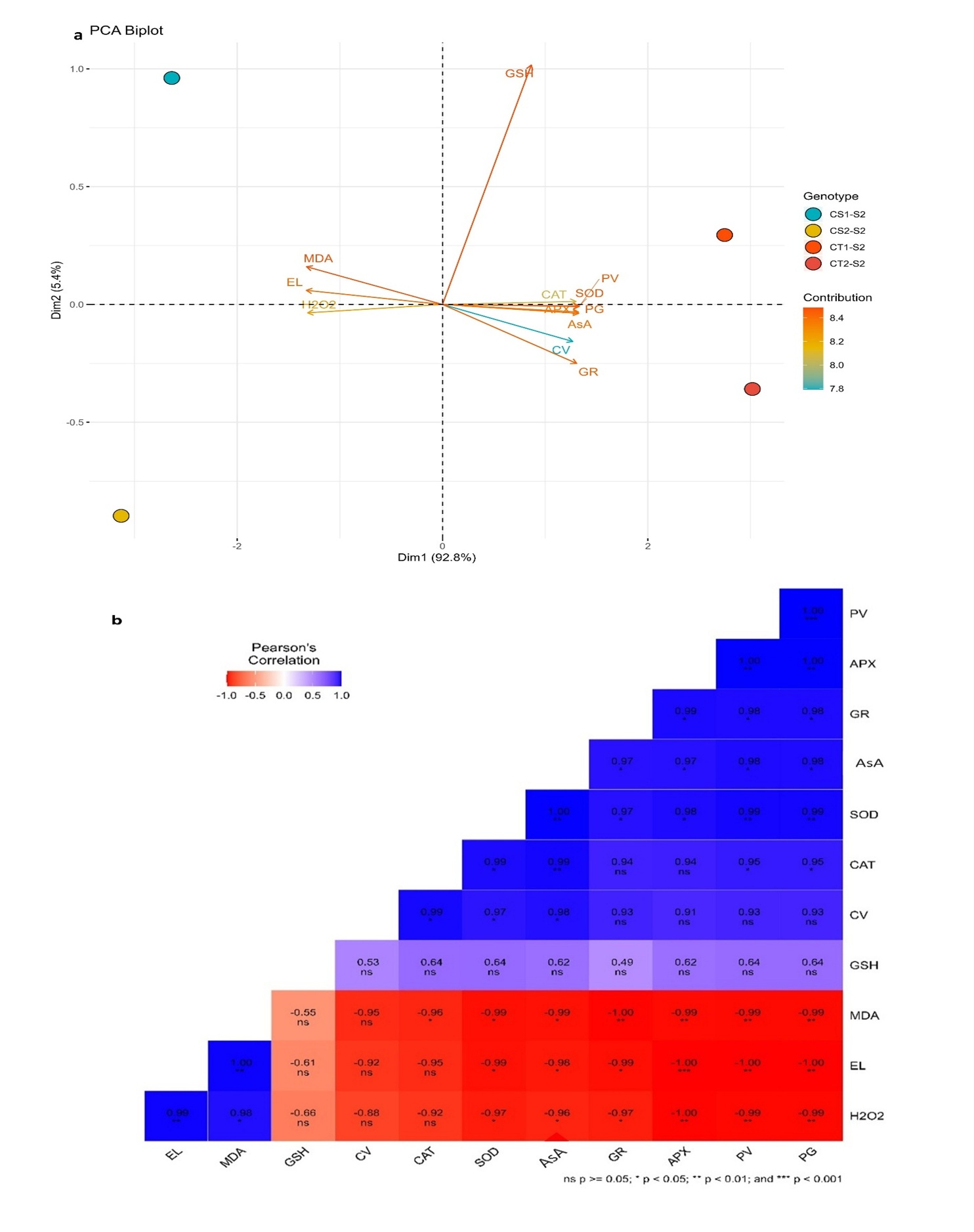


**Supplementary Fig. S5:** (a) Principal component analysis (PCA) biplot for 12 variables under cold stress at stage 2 (anthesis) of anther developmental, and (b) Pearson correlation matrix. EL: electrolyte leakage; CV: cellular viability; MDA: malondialdehyde content; H_2_O_2_: hydrogen peroxide; SOD: superoxide dismutase; CAT: catalase; APX: ascorbate peroxidase; GR: glutathione reductase; AsA: ascorbic acid; GSH: reduced glutathione; PV: pollen viability; PG: pollen germination.CT1: Cold tolerant genotype 1 (ICC 17258); CT2: Cold tolerant genotype 2 (ICC 16349); CS1: Cold sensitive genotype 1 (ICC 15567); CS2: Cold sensitive genotype 2 (GPF-2). S2-Stage 2.


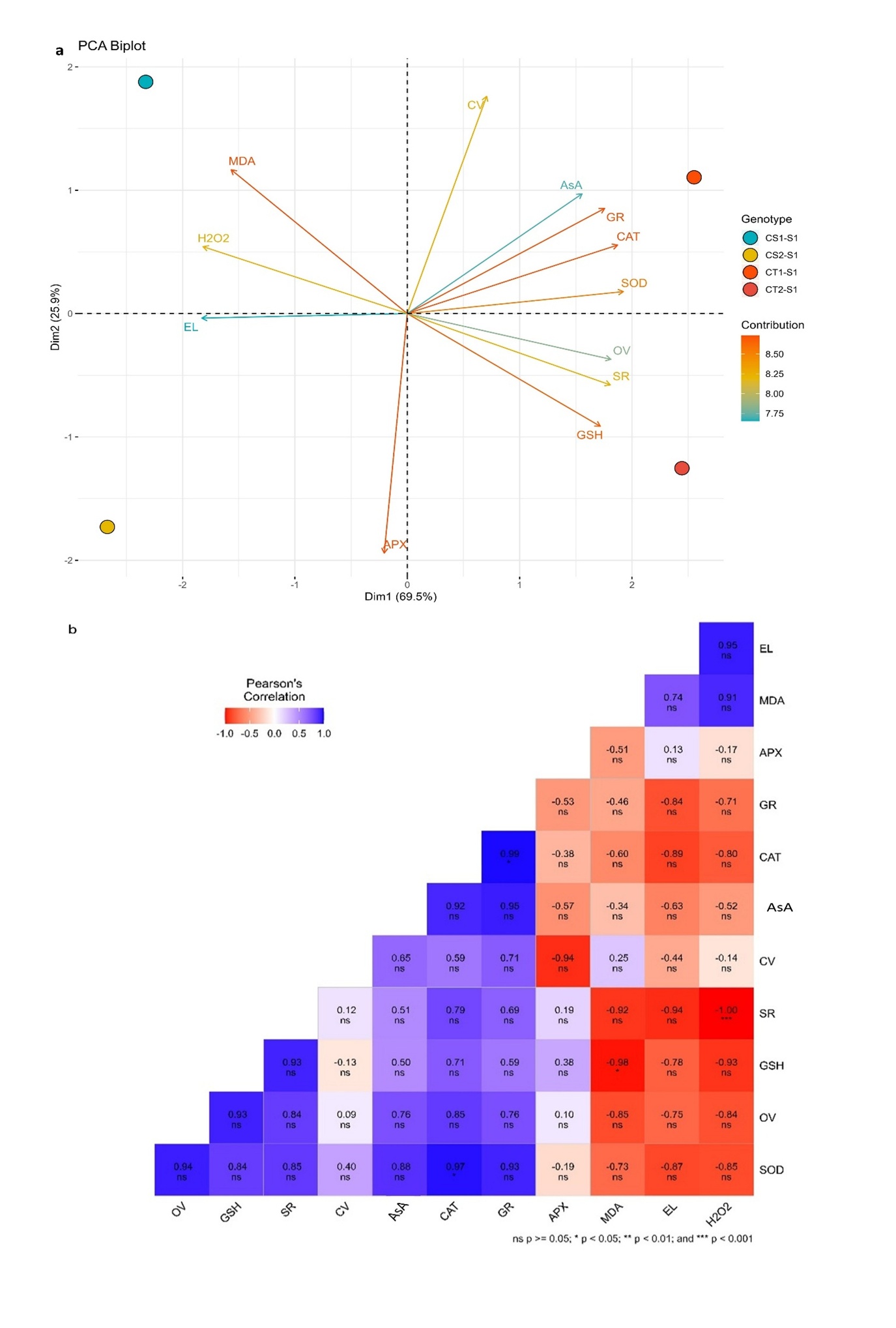


**Supplementary Fig. S6:** (a) Principal component analysis (PCA) biplot for 12 variables under cold stress at stage 1 (pre-anthesis) ovule developmental, and (b) Pearson correlation matrix. EL: electrolyte leakage; CV: cellular viability; MDA: malondialdehyde content; H_2_O_2_: hydrogen peroxide; SOD: superoxide dismutase; CAT: catalase; APX: ascorbate peroxidase; GR: glutathione reductase; AsA: ascorbic acid; GSH: reduced glutathione; PV: pollen viability; PG: pollen germination. CT1: Cold tolerant genotype 1 (ICC 17258); CT2: Cold tolerant genotype 2 (ICC 16349); CS1: Cold sensitive genotype 1 (ICC 15567); CS2: Cold sensitive genotype 2 (GPF-2). S1-Stage 1.

**
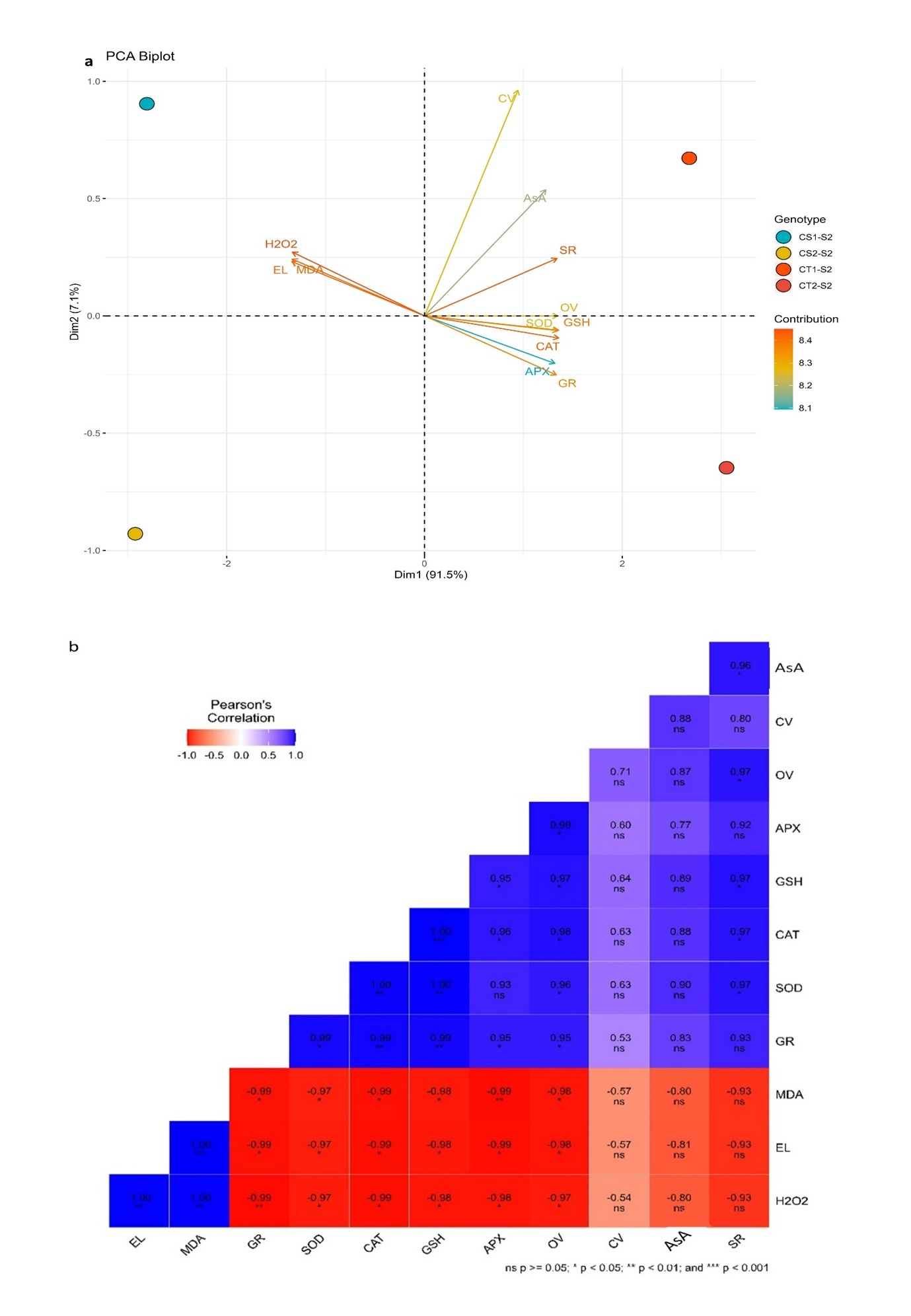
**

**Supplementary Fig. S7:** (a) Principal component analysis (PCA) biplot for 12 variables under cold stress at stage 2 (anthesis) of ovule development, and (b) Pearson correlation matrix. EL: electrolyte leakage; CV: cellular viability; MDA: malondialdehyde content; H_2_O_2_: hydrogen peroxide; SOD: superoxide dismutase; CAT: catalase; APX: ascorbate peroxidase; GR: glutathione reductase; AsA: ascorbic acid; GSH: reduced glutathione; PV: pollen viability; PG: pollen germination. CT1: Cold tolerant genotype 1 (ICC 17258); CT2: Cold tolerant genotype 2 (ICC 16349); CS1: Cold sensitive genotype 1 (ICC 15567); CS2: Cold sensitive genotype 2 (GPF-2). S2-Stage 2.


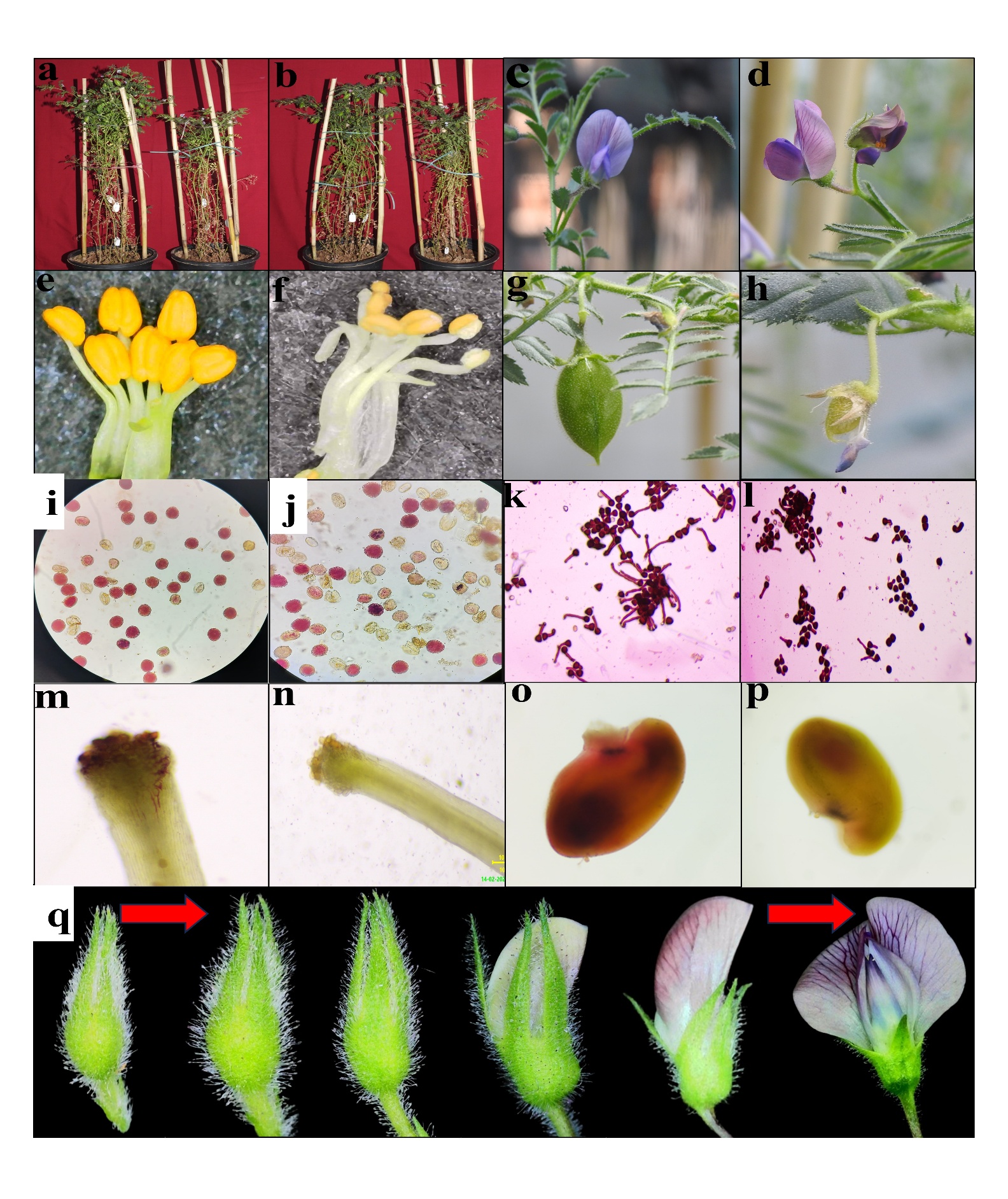


**Supplementary Fig. S8:** Effects of cold stress on chickpea. Contrasting genotypes (a) Cold tolerant 17258 (left) and Cold sensitive ICC 15667 (right) and (b) Cold tolerant 16349 (left) and Cold sensitive GPF2 (right); (c) healthy flower in CT genotype; (d) aborted flower with exposed anthers in CS genotype; (e) healthy anthers in CT genotype; (f) damaged anthers in CS genotype; (g) healthy pod in CT genotype; (h) aborted pod in CS genotype; (i) more pollen viability in CT genotype; (j) less pollen viability in CS genotype; (k) more pollen germination in CT genotype; (l) less pollen germination in CS genotype; (m) higher stigma receptivity in CT genotype; (n) lower stigma receptivity in CS genotype; (o) more ovule viability in CT genotype; (p) less ovule viability in CS genotype; (q) flower developmental stages, with stage 1 (pre-Anthesis, bud size 5 mm) and stage 2 (anthesis, flower size 10 mm) marked with red arrows.
